# Sequential clearance of neutrophil extracellular traps for precision therapy of sepsis

**DOI:** 10.1126/sciadv.aef3930

**Published:** 2026-09-02

**Authors:** Nan Wang, Yuqin Jin, Jian Song, Miaomiao Fei, Tan Wu, Hui Zhang, Changwei Wei, Qian Chen, Cheng Li

**Affiliations:** ^1^ Department of Anesthesiology and Perioperative medicine, Shanghai Key Laboratory of Anesthesiology and Brain Functional Modulation, Clinical Research Center for Anesthesiology and Perioperative Medicine, Translational Research Institute of Brain and Brain-Like Intelligence, Shanghai Fourth People’s Hospital, School of Medicine, Tongji University, Shanghai 200434, China.; ^2^ Department of Anesthesiology, Beijing Chaoyang Hospital, Capital Medical University, Beijing, China.

## Abstract

Sepsis, a life-threatening syndrome driven by dysregulated host response to infection, is critically exacerbated by the uncontrolled formation of neutrophil extracellular traps (NETs), which amplify inflammation, promote microthrombosis, and precipitate multiple organ dysfunction. However, current therapeutic approaches remain inadequate in effectively targeting and modulating NETs, leaving a significant clinical gap in managing sepsis progression. Herein, we propose a “sequential NETs-clearance” strategy to precisely mitigate NET-associated pathologies by simultaneously inhibiting NET overproduction and facilitating their targeted capture and degradation. We engineered a multifunctional metal-organic framework (MOF818) with intrinsic antioxidant activity, functionalized with a NETs-capturing poly(amidoamine) dendrimer and loaded with a NETs-degrading enzyme, micrococcal nuclease (MNase). This integrated platform efficiently scavenges excessive reactive oxygen species (ROS) to inhibit NETosis, while simultaneously capturing existing NETs via electrostatic interactions and enzymatically cleaving their DNA scaffold, thereby disrupting the self-sustaining inflammatory cascade. In vitro, this combinatorial treatment markedly reduced NET-mediated inflammatory responses, while treatment in murine models of sepsis significantly mitigated systemic cytokine storms, alleviated organ damage, and improved survival in septic mice. Importantly, this therapeutic efficacy was corroborated in ex vivo human specimens, where the nanocomposite significantly suppressed NETosis and reduced inflammatory cytokines in neutrophils and plasma derived from sepsis patients. This work establishes a versatile nanotherapeutic paradigm for multi-target modulation of NETs and oxidative stress, offering a promising translational avenue for the precise treatment of sepsis.

## INTRODUCTION

Sepsis represents a dysregulated host response to infection that can lead to life-threatening organ dysfunction (*1*, *2*). This complex syndrome is characterized by a multifaceted pathophysiology involving intertwined perturbations in the immune, inflammatory, and coagulation systems (*3*), ultimately culminating in multi-organ failure (*4*, *5*). A primary obstacle lies in the inadequacy of single-target interventions against a disorder driven by highly interconnected and often redundant pathological cascades (*6*, *7*).

A critical component of this dysregulated host response is the formation of Neutrophil Extracellular Traps (NETs) (*8*–*10*). NETs are web-like structures made of decondensed chromatin that are adorned with antimicrobial proteins like myeloperoxidase, neutrophil elastase, and histones (*11*–*13*). Through a specialized cell death process called NETosis, neutrophils release them, acting as a vital defense mechanism to capture and kill pathogens (*14*, *15*). The production of reactive oxygen species (ROS) by NADPH oxidase is essential for the start of NETosis (*16*–*18*). However, in the context of sepsis, this otherwise protective response becomes excessive and detrimental (*19*, *20*). Aberrant NET formation contributes directly to tissue injury via the cytotoxic effects of associated histones and proteases (*21*), which damage endothelial cells, disrupt vascular integrity, and promote microthrombosis (*22*), thereby exacerbating organ ischemia and dysfunction (*23*, *24*). Moreover, ROS not only promote NETosis but also function as important signaling molecules that activate key signaling pathways such as the NF-κB and MAPK pathways, thereby increasing the production of pro-inflammatory cytokines (such as TNF-α, IL-1β, and IL-6) and sustaining a vicious cycle of oxidative stress and inflammation. As a result, key treatment areas in sepsis research include the strategic control of NETosis and the subsequent clearance of NETs (*25*, *26*).

Under physiological conditions, the disassembly of NETs is primarily mediated by the serum endonuclease DNase I, which cleaves the DNA backbone, thereby preventing persistent inflammation and thrombosis (*27*). Unfortunately, this homeostatic clearance mechanism is frequently compromised in sepsis patients due to the presence of endogenous DNase I inhibitors (*28*). The exploration of alternative nucleases, such as microbial nucleases (MNases), has been pursued to overcome this limitation. However, the therapeutic application of free MNase is hampered by its potential instability under the challenging physiological conditions of sepsis, including pH variations and protease-rich environments, which can significantly curtail its enzymatic efficacy and reliability (*29*). Consequently, a robust and protective delivery platform capable of stabilizing nucleases while concurrently mitigating ROS-mediated NET formation is urgently needed.

Metal-organic frameworks (MOFs), materials made of metal clusters and organic linkers (*30*, *31*), have recently emerged as promising nanoplatforms for biomedical applications owing to their high porosity (*32*), tunable surface chemistry (*33*), and inherent catalytic or antioxidant properties (*34*, *35*). In particular, the Cu/Zr-based MOF818 exhibits strong redox activity and excellent structural stability (*36*), enabling efficient ROS scavenging and protection of sensitive biomolecules under physiological conditions (*37*, *38*). These features make MOF818 an ideal scaffold for constructing multifunctional nanotherapeutics tailored for inflammatory diseases.

**Fig. 1.**
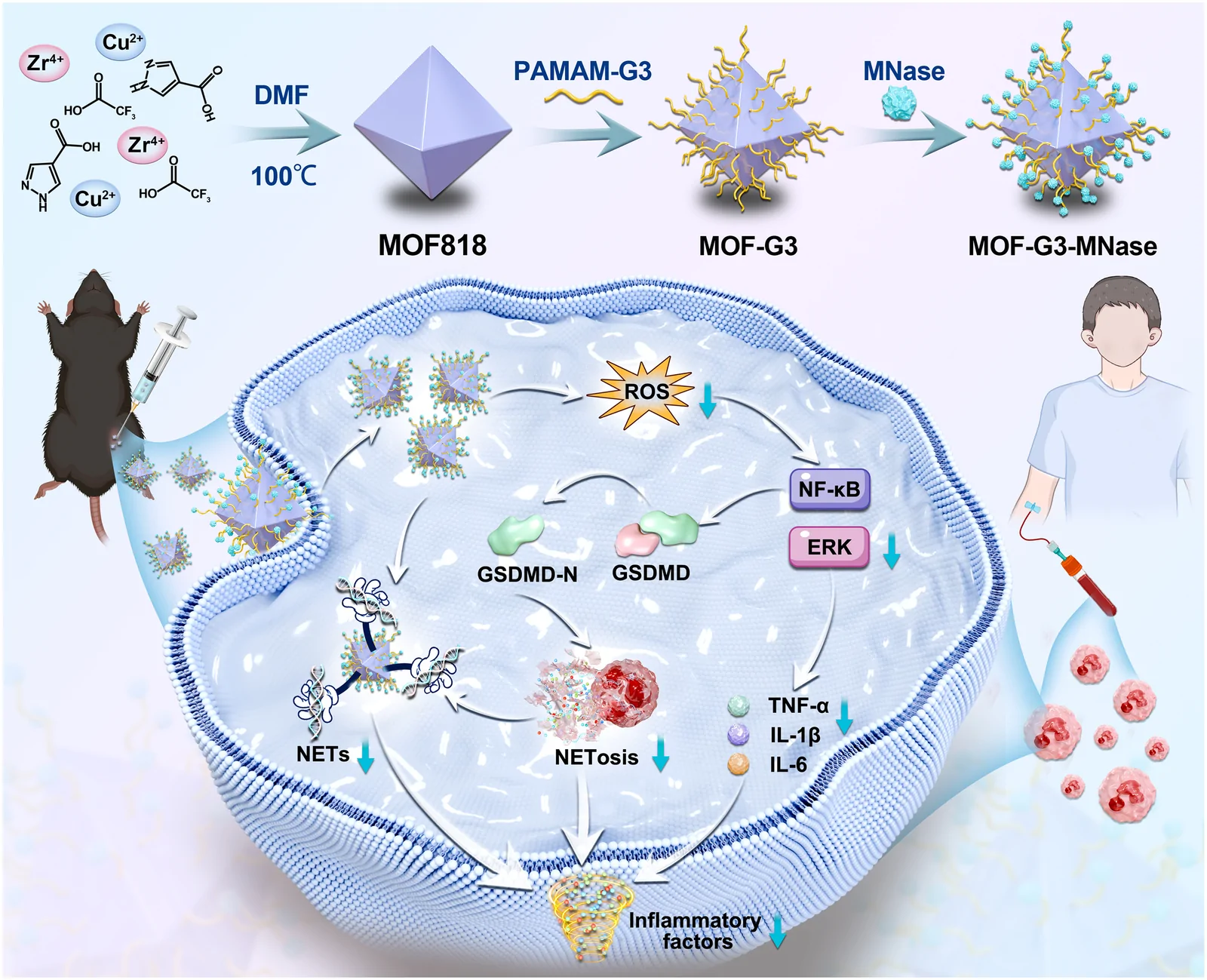
Schematic illustration of the preparation of the MOF-G3-MNase nanoparticle system and its mechanism in sepsis treatment. The MOF818 was synthesized by a facile solvothermal method. To endow it with specific biological functionalities, poly(amidoamine) dendrimer generation 3 (PAMAM-G3) and the neutrophil extracellular trap (NET)-degrading enzyme micrococcal nuclease (MNase) were sequentially functionalized onto the MOF surface, yielding the multifunctional construct MOF-G3-MNase. This agent suppresses the NF-κB-mediated inflammatory pathway through ROS reduction, leading to the inhibited production of proinflammatory factors and diminished release of NETs. Simultaneously, PAMAM-G3 exhibits the capacity to sequester NETs and facilitate their enzymatic degradation via MNase, representing a multi-targeted therapeutic strategy for sepsis management.

To address these challenges, we propose a comprehensive “sequential NETs-clearance” strategy aimed at simultaneously curbing the overproduction of NETs and enhancing the targeted capture and degradation of existing NET structures (Fig. 1). We engineered a multifunctional nanoplatform based on a metal-organic framework (MOF818) with intrinsic antioxidant properties (*33*, *36*). This core was functionalized with a NETs-capturing poly(amidoamine) dendrimer (PAMAM-G3-NH_2_), a known scavenger of nucleic acids and nucleoprotein complexes (*39*), and loaded with the NETs-degrading enzyme, MNase. This integrated design allows the nanocomposite to: (i) scavenge excessive ROS via its MOF core to suppress the initiation of NETosis, (ii) capture formed NETs via electrostatic interactions with the surface dendrimer, and (iii) locally degrade the captured NETs through the enzymatic action of the released MNase. In vitro, this combinatorial approach reduces NET formation and NET-amplified inflammatory responses. In murine models of sepsis, it attenuates cytokine storms, preserves organ function, and improves survival. Importantly, ex vivo studies with specimens from sepsis patients demonstrate suppression of NETosis and decreased inflammatory cytokines, supporting translational potential. By concurrently targeting NET formation, capture, and clearance, this multi-targeted approach aims to disrupt the self-perpetuating cycle of inflammation and thrombosis in sepsis, offering a promising and versatile nanotherapeutic paradigm for precise intervention.

## RESULTS

### Synthesis and characterization of MOF-G3-MNase nanocomposite

**Fig. 2.**
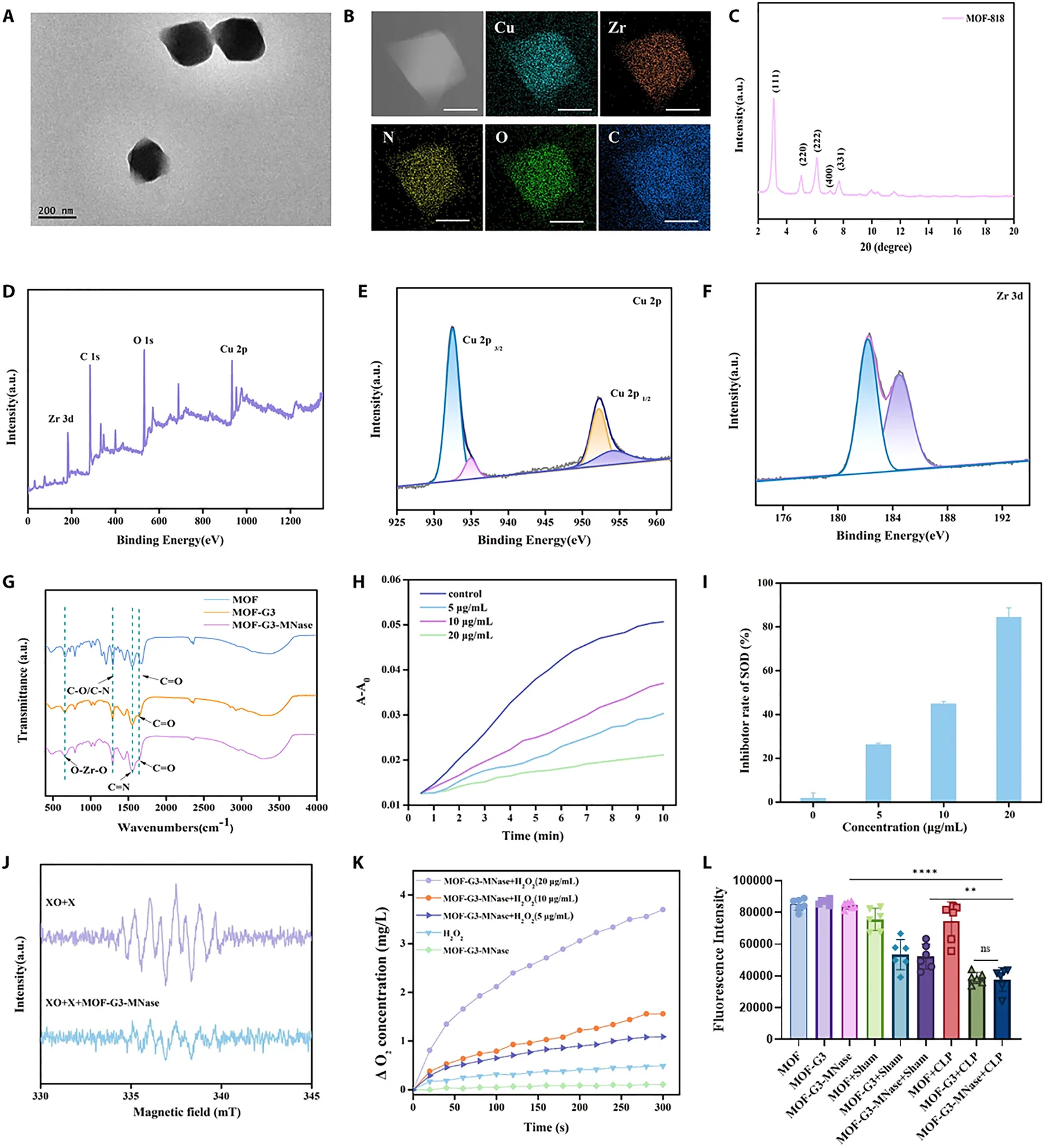
Physicochemical characterization and antioxidant performance of MOF-G3-MNase. (**A**) TEM. (**B**) Elemental mapping. (**C**) X-ray diffraction (XRD). (**D** to **F**) X-ray photoelectron spectroscopy (XPS): survey spectrum (D), high-resolution Cu 2p spectrum (E), and high-resolution Zr 3d spectrum (F). (**G**) FTIR spectrum. (**H**) SOD-like activity of MOF-G3-MNase determined using a total superoxide dismutase (SOD) activity assay based on nitroblue tetrazolium (NBT) reduction. (**I**) Superoxide anion scavenging efficiency of MOF-G3-MNase determined by the NBT assay. (**J**) Electron paramagnetic resonance (EPR) spectra showing superoxide scavenging activity of MOF-G3-MNase using the xanthine (X)/xanthine oxidase (XO) system with 5,5-dimethyl-1-pyrroline N-oxide (DMPO) as the spin-trapping agent; (**K**) Net oxygen generation from H_2_O_2_. (**L**) Fluorescence intensity in the supernatant of different groups of Cy 5.5 labeled nanomaterials co cultured with neutrophils. The results are expressed as mean ± SEM.

MOF818 was synthesized by a facile solvothermal method. To endow it with specific biological functionalities, poly(amidoamine) dendrimer generation 3 (PAMAM-G3) and the neutrophil extracellular trap (NET)-degrading enzyme micrococcal nuclease (MNase) were sequentially functionalized onto the MOF surface, yielding the multifunctional construct MOF-G3-MNase. Transmission electron microscopy (TEM) images exhibited uniform morphology and well-defined nanostructures, indicating that the subsequent surface functionalization did not disrupt the original framework (Fig. 2A). The survey spectrum (fig. S3) revealed the presence of Cu, O, C, and Zr, consistent with elemental mapping (Fig. 2B). The crystallinity of the prepared MOF818 was further validated by x-ray diffraction (XRD). As shown in Fig. 2C, the diffraction peaks of the synthesized MOF818 were in excellent agreement with the simulated standard pattern, confirming successful formation of the target framework. To further analyze the elemental composition and chemical states, x-ray photoelectron spectroscopy (XPS) was performed. High-resolution spectra displayed characteristic Cu 2p peaks at 952.4 eV and 932.5 eV, corresponding to Cu 2p_1/2_ and Cu 2p_3/2_, respectively, along with well-defined Zr 3d signals (Fig. 2, D to F). The C 1s and O 1s spectra (figs. S3 and S4) exhibited peaks attributable to C=O and C-O bonds derived from the 1H-pyrazole-4-carboxylic acid (H_2_PyC) ligand. Fourier-transform infrared (FTIR) spectroscopy provided additional confirmation of the coordination environment and ligand structure. The characteristic Zr-O-Zr stretching vibration appeared at 664 cm^−1^, while peaks at 1450 cm^−1^ and 1294 cm^−1^ were assigned to pyrazole ring skeletal vibrations and C-N/C-O stretching, respectively. The absorption bands at 1553 cm^−1^ and 1675 cm^−1^ corresponded to the C=N stretching of the pyrazole ring and the C=O stretching of -COOH groups. Importantly, PAMAM-G3 exhibited a distinct amide C=O stretching vibration at 1640 cm^−1^, a feature that was preserved in the MOF-G3 spectrum (Fig. 2G), providing direct evidence of successful amide bond formation and surface modification. As shown in Fig. 2, H and I, after the addition of MOF-G3-MNase, the signal intensity at 560 nm significantly decreased in a dose-dependent manner, indicating that MOF-G3-MNase has SOD-like activity. In addition, the CAT-like activity of MOF-G3-MNase was evaluated by monitoring the production of O_2_. As shown in Fig. 2K, MOF-G3-MNase effectively decomposes H_2_O_2_ into O_2_, and the catalytic rate increases with the addition of MOF-G3-MNase. EPR spectroscopy was also used to evaluate the ·O_2_^−^ scavenging ability of MOF-G3-MNase, with ·O_2_^−^ detected by 5,5 - dimethyl-1-pyrroline-1-oxide (DMPO). As expected, MOF-G3-MNase effectively removes ·O_2_^−^, as shown by the decrease in EPR amplitude of DMPO-OOH (Fig. 2J). The NET-targeting ability of MOF-G3-MNase was assessed in vitro. Twenty-four hours hours after modeling CLP induction, bone marrow neutrophils were isolated from control and CLP mice and co-incubated with Cy5.5-labeled MOF818, MOF-G3, or MOF-G3-MNase. Quantitative fluorescence analysis after filtering out NETs showed that the binding and accumulation of PAMAM-G3 modified nanoplatforms at NETs sites were significantly enhanced (Fig. 2L). These results confirm that PAMAM-G3 functionalization markedly improves the targeting specificity of the nanoplatform toward NET structures, a critical feature for subsequent therapeutic applications.

### MOF-G3-MNase nanoplatform suppresses NETosis, mitigates ROS, and attenuates systemic inflammation in sepsis

**Fig. 3.**
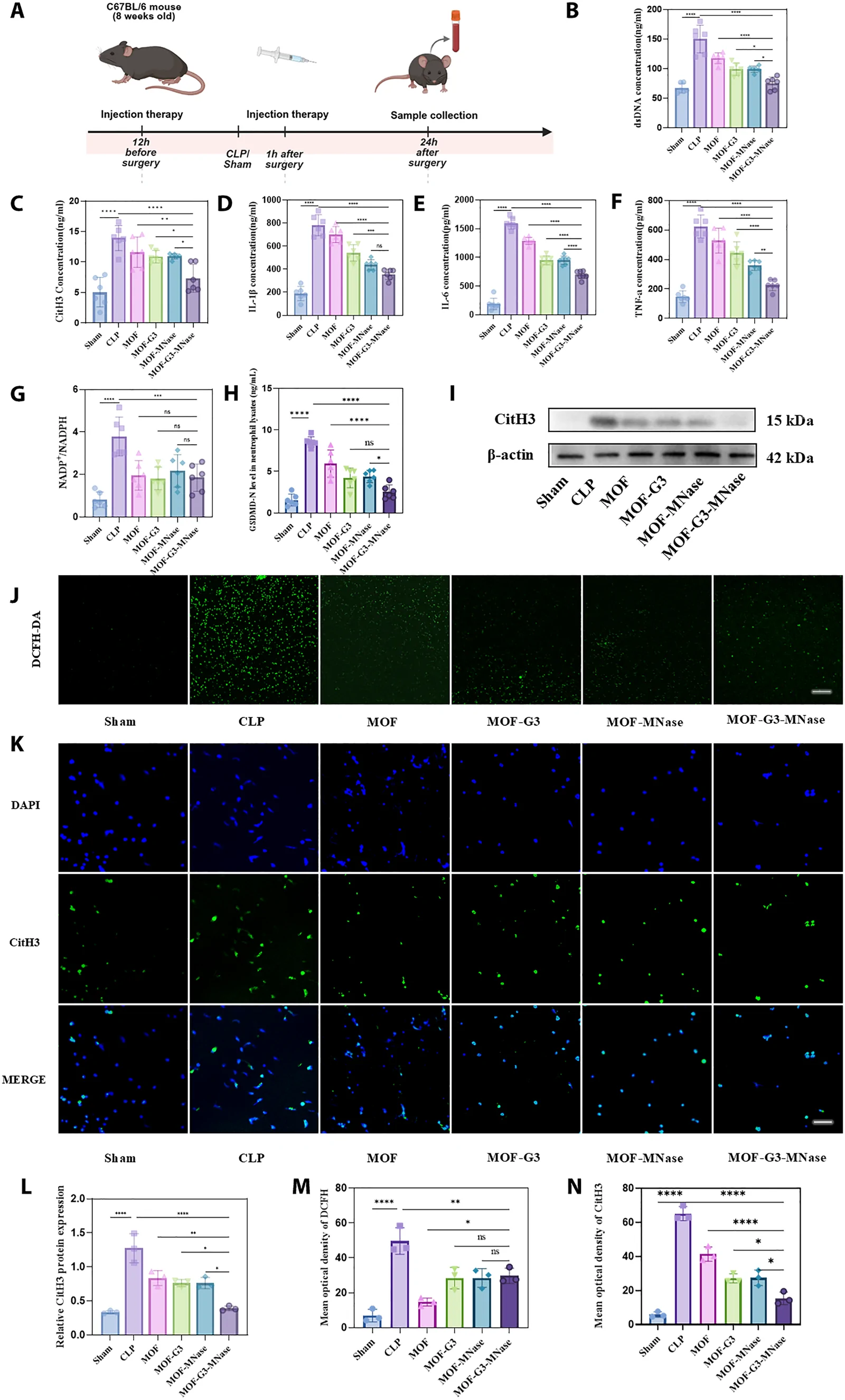
MOF-G3-MNase reduces NETs, inflammatory factors, and ROS in septic mice. (**A**) Schematic of CLP model establishment and treatment schedule. Nanoparticles were administered at 20 mg/kg 12 h before and 1 h after CLP surgery, and samples were collected 24 h postoperatively. (**B** and **C**) Plasma dsDNA and CitH3 levels were measured by PicoGreen assay and ELISA, respectively (n = 6 per group). (**D** to **F**) Plasma IL-1β, IL-6, and TNF-α levels were determined by ELISA (n = 6 per group). (**G**) NADPH oxidase activity was assessed using the WST-8 method based on the NADP+/NADPH ratio (n = 6 per group). (**H**) GSDMD-N levels in mouse neutrophils were measured by ELISA (n = 6 per group). (**I** and **L**) CitH3 expression in neutrophils was analyzed by Western blotting and quantified (n = 3 per group). (**J** and **M**) Intracellular ROS levels were visualized by DCFH-DA staining and quantified by fluorescence intensity. Scale bar, 50 μm (n = 3 per group). (**K** and **N**) NET formation was assessed by immunofluorescence staining and quantified based on CitH3 fluorescence intensity. Scale bar, 200 μm (n = 3 per group). Data are presented as mean ± SEM. Statistical analysis was performed using one-way ANOVA followed by Tukey’s post hoc test. (A) Created in BioRender. Z. Yy (2026) https://BioRender.com/ldsukkk.

We first evaluated the biosafety of the MOF-G3-MNase nanoplatform. After administration to healthy mice, we detected plasma biochemical markers including AST, ALT, BUN and CREA at 24 hours and 30 days post-treatment, and performed H&E staining to observe the histological morphology of the heart, liver, spleen, lung and kidney. No obvious abnormalities were found in physiological indices or tissue structures compared with the control group, suggesting favorable short-term and preliminary 30-day biosafety of MOF-G3-MNase under the current experimental conditions (figs. S5 to S8, S17, and S18). A murine model of polymicrobial sepsis was then established by cecal ligation and puncture (CLP) to investigate the therapeutic potential of the MOF-G3-MNase nanoplatform. Mice were intraperitoneally injected with the materials 12 h prior to and 1 h after surgery, and samples were collected 24 h post-modeling (Fig. 3A). Neutrophil extracellular traps (NETs) are chromatin–protein complexes expelled by activated neutrophils that play a dual role in antimicrobial defense and inflammation propagation. Excessive NET formation is known to exacerbate sepsis-induced tissue injury. To assess the effect of the nanoplatform on NETosis, plasma levels of double-stranded DNA (dsDNA) and citrullinated histone H3 (CitH3), two hallmark NET markers, were measured. Compared with the Sham group, both dsDNA and CitH3 were markedly elevated in septic mice but were significantly reduced upon treatment with MOF-G3-MNase. Notably, this dual-functional nanoplatform exhibited superior efficacy compared with single-component formulations (MOF, MOF-G3, or MOF-MNase) (Fig. 3, B and C). To further visualize the proposed sequential “capture-then-degrade” process, we performed time-course confocal imaging of pre-formed NETs incubated with Cy5.5-labeled MOF-G3-MNase. SYTOX Green was used to stain extracellular NET-DNA structures. At the early time point, MOF-G3-MNase rapidly colocalized with SYTOX-positive NET structures, indicating efficient NET capture. At later time points, the extracellular DNA network progressively decreased and became fragmented, accompanied by reduced SYTOX fluorescence intensity, suggesting enzymatic degradation of the NET-DNA scaffold by MNase. These findings provide direct dynamic evidence supporting the sequential NET capture and degradation mechanism of MOF-G3-MNase (fig. S19). To further compare the efficacy of MOF-G3-MNase with established NET-targeting interventions, we included DNase I and the PAD4 inhibitor GSK484 as reference treatments. MOF-G3-MNase reduced circulating dsDNA and CitH3 levels more effectively than DNase I or GSK484 under the same experimental conditions (figs. S13 to S15). Next, the levels of key pro-inflammatory cytokines (IL-6, IL-1β, and TNF-α) were determined to evaluate systemic inflammatory responses. Consistent with the NETs results, the MOF-G3-MNase group showed profound suppression of inflammatory cytokines relative to both the CLP group and other treatment groups (Fig. 3, D to F). Reactive oxygen species (ROS) play a pivotal role in triggering NETosis and inflammatory amplification. Since NADPH oxidase is a major source of intracellular ROS, we assessed its activity using the WST-8 assay based on the NADP^+^/NADPH ratio. All MOF-based nanoparticles, particularly MOF-G3-MNase, significantly reduced the NADP^+^/NADPH ratio compared with septic controls, confirming the ability of the nanomaterials to attenuate oxidative stress (Fig. 3G). Gasdermin D (GSDMD), a key executor of pyroptosis, links cytokine activation with inflammatory cell death and NETosis (*40*, *41*). Given that the ELISA kit used in the analysis detects full-length GSDMD and C-terminal-containing GSDMD species, but not the pore-forming GSDMD-N fragment, the measured value was defined as the ELISA-detectable full-length/C-terminal-containing GSDMD signal. Compared with the Sham group, this ELISA-detectable GSDMD signal was decreased in CLP mice, which may reflect proteolytic consumption of full-length GSDMD during sustained inflammatory activation (fig. S11). To directly assess GSDMD activation, GSDMD-N levels in neutrophils were further examined. CLP markedly increased GSDMD-N levels in neutrophils, whereas MOF-G3-MNase treatment significantly reduced GSDMD-N levels, indicating that MOF-G3-MNase attenuated GSDMD cleavage-associated inflammatory activation in septic neutrophils (Fig. 3H). Western blot analysis of CitH3 levels in isolated neutrophils further validated the inhibitory effect of MOF-G3-MNase on NET formation (Fig. 3, I and L). To directly assess intracellular ROS dynamics, neutrophils were stained with the DCFH-DA probe and visualized by fluorescence microscopy. Neutrophils from septic mice displayed intense green fluorescence indicative of high ROS levels, whereas those treated with MOF-G3-MNase exhibited markedly reduced fluorescence, confirming efficient ROS scavenging (Fig. 3, J and M). Immunofluorescence staining further revealed extensive chromatin decondensation and CitH3 co-localization in neutrophils from CLP mice, while cells in the MOF-G3-MNase group retained normal nuclear morphology with minimal CitH3 deposition, demonstrating that the nanoplatform effectively inhibited NETosis (Fig. 3, K and N). These results demonstrate that the engineered MOF-G3-MNase nanoplatform synergistically suppresses excessive ROS generation and NET release, thereby disrupting the self-amplifying inflammatory cascade characteristic of sepsis. To assess whether MOF-G3-MNase impaired bacterial clearance, blood bacterial burden was evaluated by CFU counting at 24 h after CLP surgery. CLP markedly increased blood CFU counts compared with the Sham group, whereas MOF-G3-MNase treatment significantly reduced blood bacterial burden compared with untreated CLP mice. These results suggest that MOF-G3-MNase attenuated excessive NET/ROS-driven inflammation without compromising systemic bacterial clearance (fig. S12).

### MOF-G3-MNase mitigates multiorgan injury and improves survival in severe sepsis

**Fig. 4.**
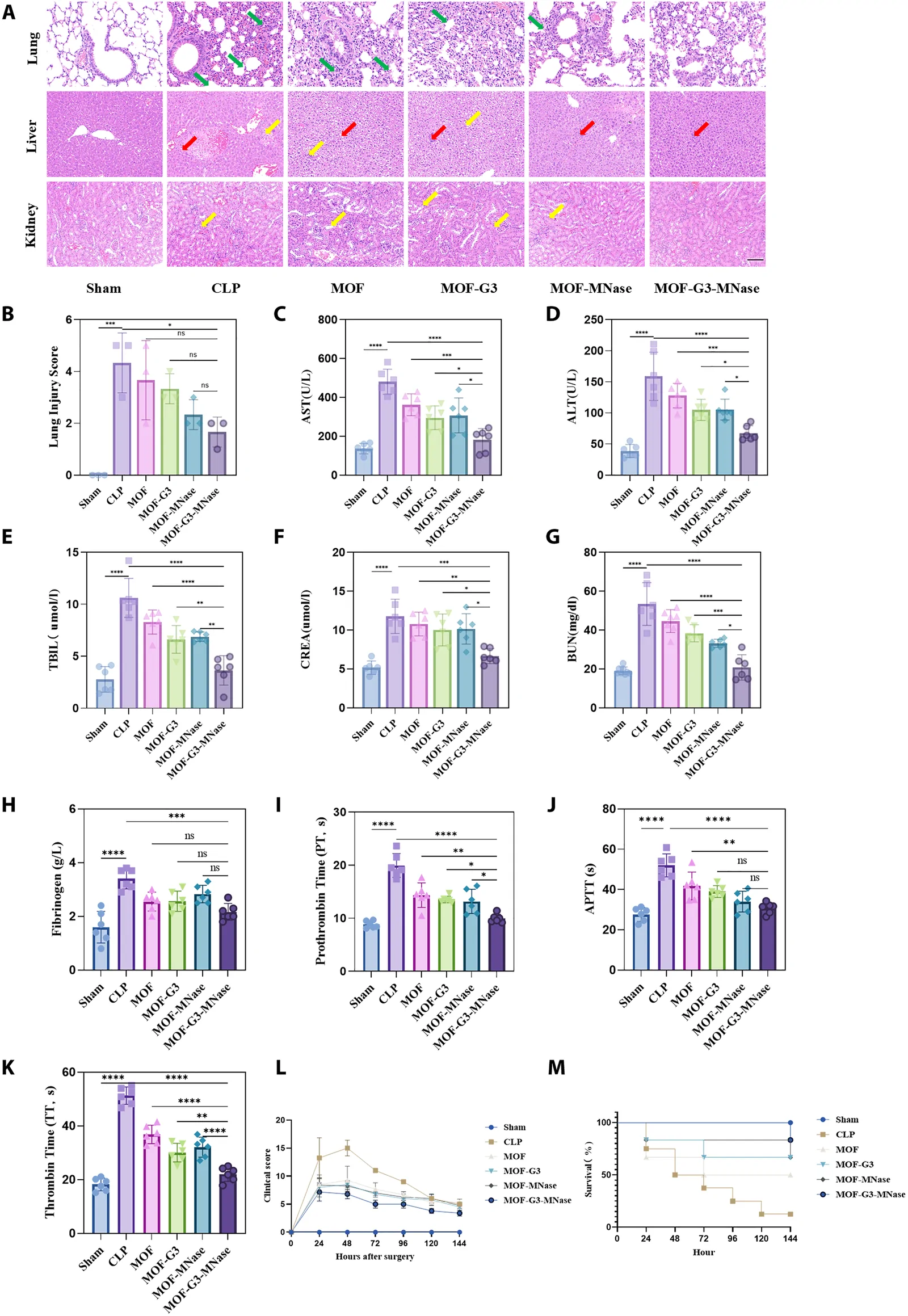
MOF-G3-MNase alleviates CLP-induced multiorgan injury and improves survival in severe sepsis. (**A**) H&E staining of lung, liver, and kidney tissues collected 24 h after CLP. Scale bar, 50 μm. Green arrows indicate inflammatory cell infiltration in the alveolar interstitium, red arrows indicate inflammatory cell aggregation forming necrotic foci, and yellow arrows indicate inflammatory cell infiltration in liver and kidney tissues (n = 3 per group). (**B**) Semi-quantitative lung injury scores based on H&E-stained sections (n = 3 per group). (**C** to **G**) Plasma AST, ALT, TBIL, CREA, and BUN levels were measured to evaluate organ injury (n = 6 per group). (**H**) Plasma fibrinogen levels at 24 h after CLP (n = 6 per group). (**I** to **K**) Plasma PT, APTT, and TT were measured to evaluate coagulation function (n = 6 per group). (**L**) Clinical scores were monitored for 144 h after CLP (n = 6 per group). (**M**) Survival was monitored for 144 h after CLP (n = 10 per group). Mice were randomly assigned to Sham, CLP, MOF818, MOF-G3, MOF-MNase, and MOF-G3-MNase groups. Nanoparticles were administered intraperitoneally at 20 mg/kg 12 h before and 1 h after CLP. Data are presented as mean ± SEM. Statistical analysis was performed using one-way ANOVA followed by Tukey’s post hoc test.

Since multiple organ failure is a major outcome of severe sepsis, histopathological and biochemical analyses of key organs were performed 24 h after CLP to further assess the in vivo therapeutic efficacy of the nanoplatform. Severe structural damage was observed in the lung, liver and kidney of septic mice, including thickened alveolar septa, diffuse inflammatory cell infiltration, and hepatic and renal necrosis. In contrast, the MOF-G3-MNase group exhibited markedly preserved tissue architecture with minimal inflammatory infiltration (Fig. 4A). Semi-quantitative histopathological scoring confirmed significant reductions in tissue injury compared with the sepsis group and other treatments groups (Fig. 4B). Plasma biochemical markers further corroborated these findings. Aspartate aminotransferase (AST), alanine aminotransferase (ALT), total bilirubin (TBIL), creatinine (CREA), and blood urea nitrogen (BUN) levels were significantly elevated in septic mice but were markedly decreased following MOF-G3-MNase treatment (Fig. 4, C to G). Notably, significantly elevated plasma FIB levels in the CLP group indicated that mice at 24 h post-CLP were predominantly subjected to acute inflammatory-driven hypercoagulability rather than severe consumptive disseminated intravascular coagulation (DIC). Treatment with MOF-G3-MNase markedly reduced FIB concentrations, restoring them toward sham-group levels (Fig. 4H). Pronounced prolongation of PT in the CLP group reflected impaired function of the extrinsic coagulation cascade, whereas PT was significantly shortened and normalized in the MOF-G3-MNase-treated group, strongly supporting ameliorated coagulation homeostasis (Fig. 4I). Similarly, prolonged APTT in CLP mice revealed disruption of the intrinsic coagulation pathway, which was effectively reversed following MOF-G3-MNase intervention (Fig. 4J). TT was substantially prolonged in septic mice, yet showed a striking reduction upon MOF-G3-MNase treatment. This observation is particularly insightful, as it demonstrates the restoration of aberrant fibrin formation processes (Fig. 4K). Clinical scoring over 144 h revealed improved physiological status in the MOF-G3-MNase group, accompanied by a striking enhancement in overall survival rate compared with control and single-component formulations (Fig. 4, L and M). These results collectively confirm that MOF-G3-MNase not only suppresses NETosis and systemic inflammation but also provides robust protection against multi-organ failure, ultimately improving the survival outcome in severe sepsis. The therapeutic effect likely arises from the synergistic actions of ROS scavenging, NET capture, and enzymatic degradation, which together re-establish immune homeostasis and prevent inflammatory overdrive. To further evaluate the therapeutic efficacy of MOF-G3-MNase after sepsis induction, we performed an additional post-treatment experiment in which MOF-G3-MNase was administered only at 1 h after CLP surgery. The post-treatment regimen significantly reduced circulating NET-associated markers, including dsDNA and CitH3, decreased inflammatory cytokines, including TNF-α and IL-6, and improved clinical scores and survival compared with untreated CLP mice. Although the original two-dose regimen produced a stronger protective effect, the post-treatment results demonstrate that MOF-G3-MNase remains therapeutically effective when administered after CLP induction (figs. S8 to S10). To further characterize the in vivo behavior of MOF-G3-MNase, we analyzed its blood concentration profile and tissue biodistribution after intraperitoneal injection. ICP-OES analysis showed that blood Zr concentrations gradually increased during the early post-injection phase, reached a peak within several hours, and subsequently declined over time, indicating systemic circulation followed by progressive clearance (fig. S20). Biodistribution analysis demonstrated that Zr signal was mainly detected in the spleen, liver, and lung, whereas relatively low accumulation was observed in the heart and kidney (fig. S21). These findings indicate that MOF-G3-MNase exhibits prolonged circulation and preferential distribution in reticuloendothelial-system-associated organs.

### Validation of MOF-G3-MNase nanoplatform using patient-derived neutrophils and plasma

**Fig. 5.**
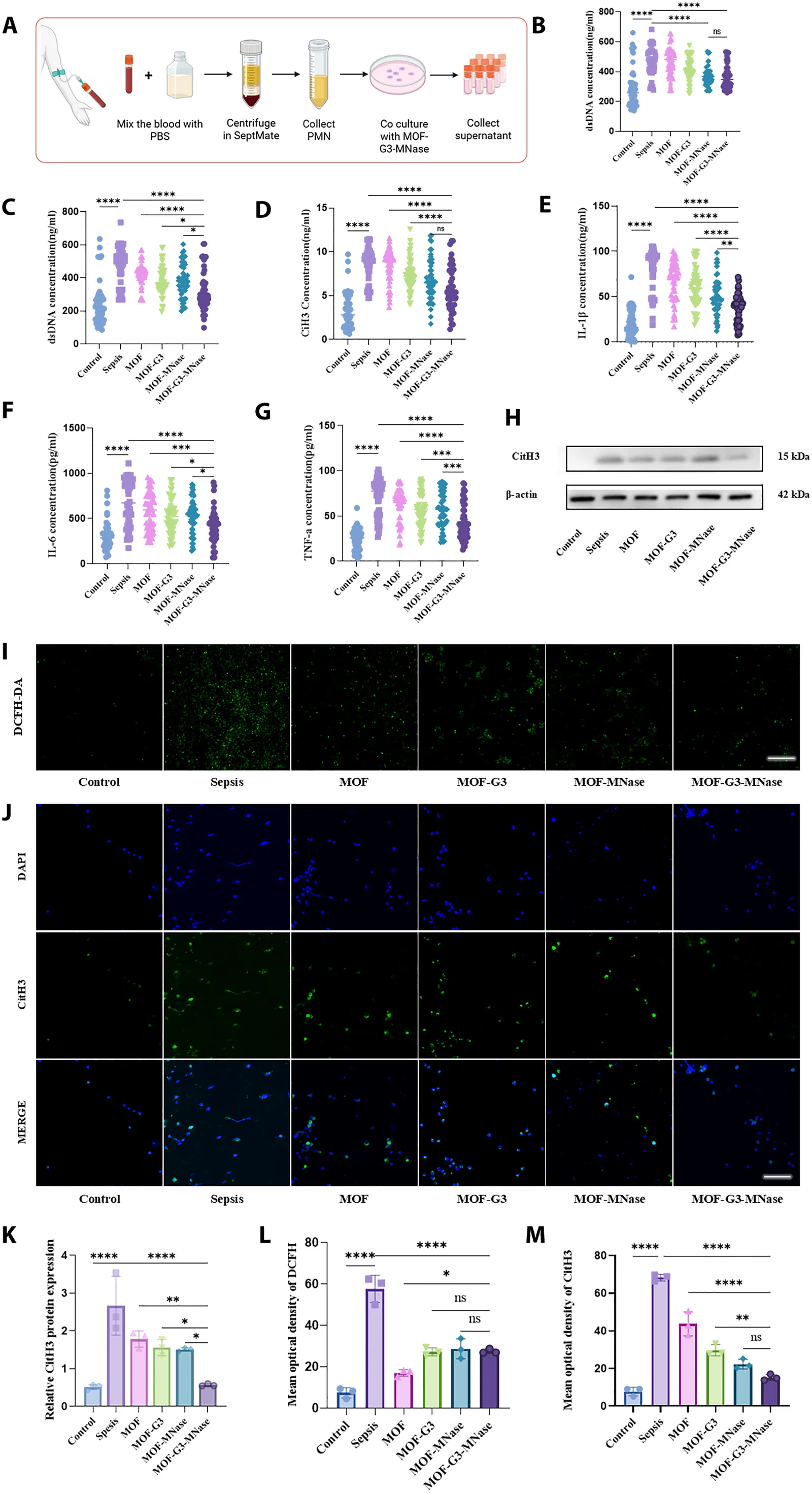
MOF-G3-MNase reduces NETs, inflammatory factors, and ROS in vitro in samples derived from patients with sepsis. (**A**) Schematic of plasma and neutrophil isolation from patients with sepsis and the in vitro treatment protocol. (**B**) dsDNA levels in patient plasma were measured by PicoGreen assay (n = 54 per group). (**C** and **D**) dsDNA and CitH3 levels in neutrophil culture supernatants were determined by PicoGreen assay and ELISA, respectively (n = 54 per group). (**E** to **G**) IL-1β, IL-6, and TNF-α levels in neutrophil culture supernatants were measured by ELISA (n = 54 per group). (**H** and **K**) CitH3 expression in neutrophils was analyzed by Western blotting and quantified (n = 3 per group). (**I** and **L**) Intracellular ROS levels were visualized by DCFH-DA staining and quantified by fluorescence intensity. Scale bar, 50 μm (n = 3 per group). (**J** and **M**) NET formation was assessed by immunofluorescence staining and quantified based on CitH3 fluorescence intensity. Scale bar, 200 μm (n = 3 per group). Patient-derived plasma or neutrophils were incubated with PBS, MOF818, MOF-G3, MOF-MNase, or MOF-G3-MNase for 3 h at an equivalent nanoparticle concentration of 20 μg/ml. Data are presented as mean ± SEM. Statistical analysis was performed using one-way ANOVA followed by Tukey’s post hoc test. (A) Created in BioRender. Z. Yy (2026) https://BioRender.com/nsa34ge.

To further verify the translational potential of the MOF-G3-MNase nanoplatform, we conducted in vitro assays using neutrophils and plasma isolated from sepsis patients. Following ethical approval, peripheral blood was collected in anticoagulant tubes. Plasma was separated by centrifugation, and neutrophils were isolated by discontinuous density gradient centrifugation. The isolated samples were co-incubated with various nanoparticles for 3 h, and multiple NETs and inflammation-associated biomarkers were quantitatively assessed (Fig. 5A). Compared with untreated sepsis plasma, treatment with MOF-MNase and MOF-G3-MNase markedly reduced the levels of extracellular dsDNA, a key marker of NETs formation (Fig. 5B), demonstrating that the MNase enzyme immobilized within MOF-G3-MNase nanoplatform retains its bioactivity and effectively degrades extracellular DNA scaffolds of NETs in patient plasma. When human neutrophils were co-cultured with different nanoparticles, both dsDNA and citrullinated histone H3 (CitH3) in the supernatants were substantially reduced in the MOF-G3-MNase group compared with the control, MOF, MOF-G3, and MOF-MNase groups (Fig. 5, C and D). This synergistic effect can be attributed to the dual functionality of MOF-G3-MNase, wherein PAMAM-G3 facilitates electrostatic capture of NETs while MNase mediates enzymatic degradation, leading to more complete NETs clearance. In parallel, the levels of pro-inflammatory cytokines IL-6, IL-1β, and TNF-α were markedly suppressed in the MOF-G3-MNase group, indicating effective alleviation of inflammatory activation (Fig. 5, E to G). Western blot analysis further confirmed a pronounced decrease in intracellular CitH3 expression following MOF-G3-MNase treatment (Fig. 5, H and K), supporting the conclusion that the MOF-G3-MNase nanoplatform efficiently inhibits NETosis and accelerates NETs degradation in sepsis-derived neutrophils. Reactive oxygen species (ROS) generation, a key trigger of NETosis, was evaluated using the fluorescent probe DCFH-DA. Fluorescence imaging revealed that all nanoparticle formulations mitigated ROS accumulation to varying degrees, with the most significant reduction observed in the MOF-G3-MNase group (Fig. 5, I and L). Immunofluorescence staining of neutrophils further visualized extensive NET formation and irregular nuclear morphology in sepsis samples, characterized by strong CitH3-positive signals. In contrast, cells treated with MOF-G3-MNase exhibited restored nuclear integrity and minimal CitH3 deposition (Fig. 5, J and M). Collectively, these results demonstrate that MOF-G3-MNase effectively suppresses ROS production, inhibits NETosis, and promotes enzymatic degradation of preformed NETs in ex vivo specimens from sepsis patients. This dual-mode mechanism—combining antioxidant capacity with NET clearance—highlights the potential of the nanocomposite as a potentially translatable therapeutic strategy for sepsis management. Although several individual components showed partial effects, the complete MOF-G3-MNase formulation produced the most consistent and significant reductions across extracellular DNA, CitH3, ROS, and cytokine readouts, supporting the cooperative contribution of extracellular NET targeting and inflammatory modulation.

### Transcriptome reprogramming induced by MOF-G3-MNase in sepsis treatment

**Fig. 6.**
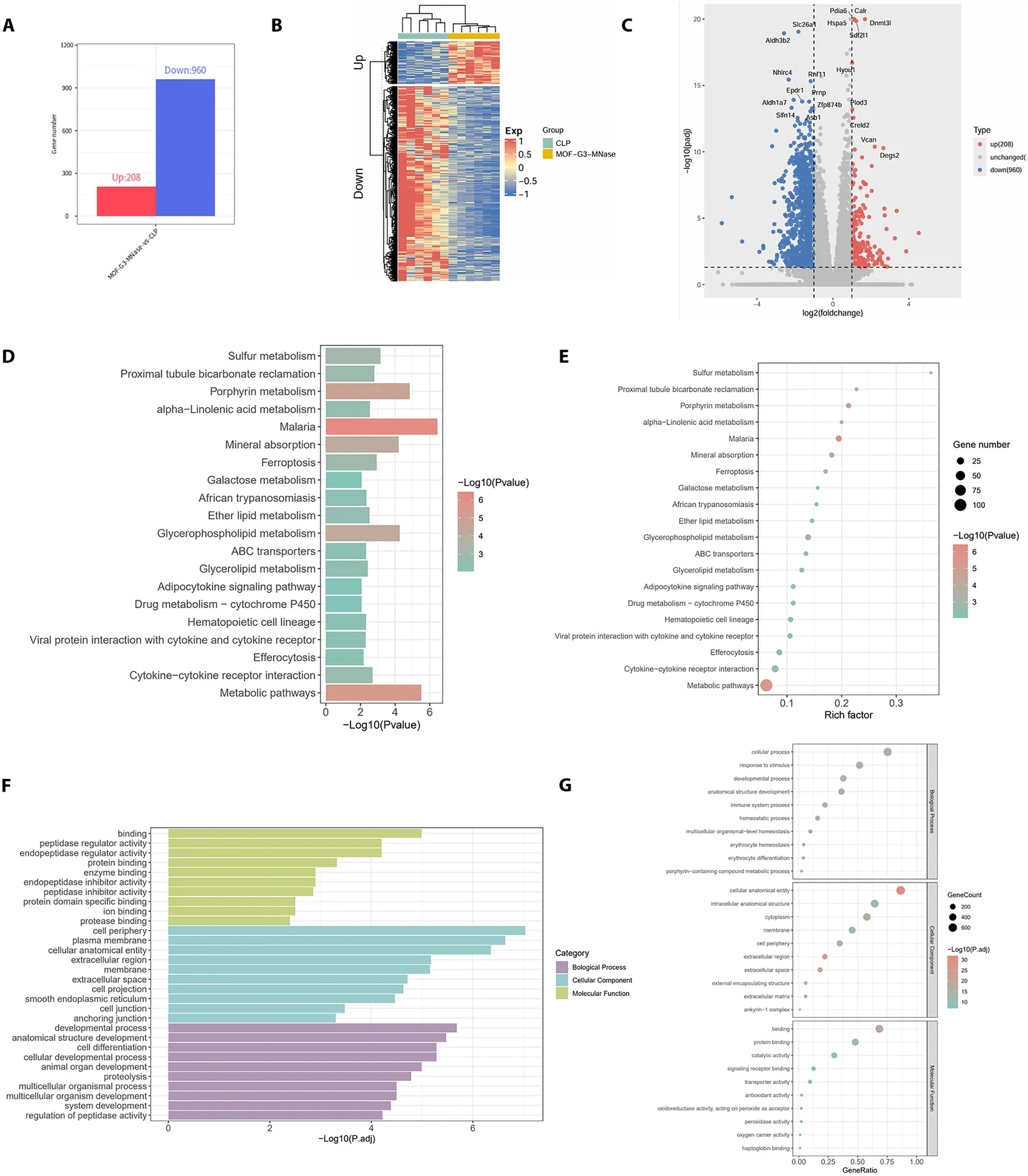
Transcriptomic analysis of neutrophils from septic mice following MOF-G3-MNase treatment. (**A**) Differential gene expression profiles in MOF-G3-MNase vs. CLP groups; (**B**) Heatmap of differentially expressed genes in neutrophils from the CLP and MOF-G3-MNase groups; (**C**) Differential gene expression analysis between CLP and MOF-G3-MNase groups; (**D** and **E**) KEGG pathway enrichment analysis of DEGs; (**F** and **G**) GO enrichment analysis of DEGs. Mice were randomly divided into CLP and MOF-G3-MNase groups; bone marrow neutrophils were isolated 24 hours after CLP (n = 6 mice per group).

To further elucidate the molecular mechanism underlying the therapeutic efficacy of MOF-G3-MNase, we performed RNA sequencing (RNA-seq) on bone marrow neutrophils isolated from CLP mice with or without MOF-G3-MNase treatment. To clarify the directionality of pathway changes, differentially expressed genes between the CLP and MOF-G3-MNase groups were divided into up-regulated and down-regulated subsets before enrichment analysis. GO and KEGG enrichment analyses were then performed separately for the up-regulated and down-regulated DEGs. This analysis revealed distinct biological processes and pathways associated with genes increased or decreased after MOF-G3-MNase treatment, allowing the direction of transcriptional changes to be interpreted more clearly. The numbers of differentially expressed genes between the CLP and MOF-G3-MNase groups are shown in Fig. 6A. Compared with the CLP group, MOF-G3-MNase treatment resulted in 208 up-regulated and 860 down-regulated genes (Fig. 6A). Comparative transcriptomic analysis showed profound transcriptional reprogramming following MOF-G3-MNase treatment. Compared with the CLP (control) group, 1,168 differentially expressed genes (DEGs) were identified, including 208 up-regulated and 960 down-regulated transcripts, while 27,991 genes remained unchanged (Fig. 6, B and C). The predominance of down-regulated genes suggests broad transcriptional reprogramming after MOF-G3-MNase treatment, involving immune, inflammatory, and redox-related pathways. Functionally, several up-regulated genes, including Hspa5 (heat-shock protein A5) and Dnmt3l (DNA methyltransferase 3-like), were enriched in stress response and epigenetic regulation, indicating an adaptive cytoprotective mechanism. Conversely, down-regulated genes such as Slc26a1 (sulfate transporter) and Aldh3b2 (aldehyde dehydrogenase) implied inhibition of aberrant metabolic activity during sepsis. Moreover, differential expression of Rnf11 (E3 ubiquitin ligase) and Hyou1 (endoplasmic reticulum stress-inducible protein) points to remodeling of ubiquitin-proteasome and ER-stress pathways, suggesting a shift toward homeostatic signaling networks (Fig. 6C). KEGG pathway enrichment further revealed 19 significantly altered signaling and metabolic pathways. Among these, sulfur metabolism, ferroptosis, porphyrin, and glycerophospholipid metabolism were the most prominently affected (Fig. 6, D and E). These pathways are intimately associated with redox balance and oxidative stress regulation, corroborating the antioxidant and iron-homeostatic functions of the MOF-based catalytic scaffold. Gene Ontology (GO) enrichment analysis showed that “response to stimulus” dominated the biological process category, while “extracellular matrix” and “oxidoreductase activity” were enriched in cellular component and molecular function categories, respectively (Fig. 6, F and G). Together, these findings substantiate the anti-inflammatory, antioxidative, and tissue-protective roles of MOF-G3-MNase at the transcriptomic level. Because NF-κB activation is mainly regulated by post-translational events rather than by transcript abundance alone, we further examined NF-κB p65 activation at the protein level. Western blot analysis showed that total NF-κB p65 levels was not significantly changed among groups, whereas phosphorylated NF-κB p65 was markedly increased after CLP and significantly reduced by MOF-G3-MNase treatment. These results indicate that MOF-G3-MNase attenuated NF-κB activation primarily by suppressing p65 phosphorylation rather than altering total NF-κB p65 abundance (fig. S16).

## DISCUSSION

Sepsis remains one of the most challenging clinical syndromes, driven by an imbalanced host response to infection that simultaneously provokes overwhelming inflammation and immune exhaustion. Among the key pathological mediators, neutrophil extracellular traps (NETs) have emerged as double-edged swords: while essential for pathogen control, their excessive formation promotes vascular damage, thrombosis, and organ failure. However, therapeutic modulation of NETs has remained elusive due to the intertwined regulation of reactive oxygen species (ROS)-dependent NETosis and impaired endogenous NET clearance. In this study, we established a “sequential NETs-clearance” strategy through a multifunctional nanoplatform (MOF-G3-MNase) that integrates catalytic ROS scavenging, NET capture, and enzymatic degradation within a single construct. This rational design directly addresses two critical bottlenecks in sepsis therapy: uncontrolled oxidative stress and persistent NET accumulation. The antioxidant MOF818 core effectively scavenged excess ROS and reduced the NADP⁺/NADPH ratio, thereby suppressing the initiation of NETosis. Meanwhile, the surface-anchored PAMAM-G3 dendrimer electrostatically bound NET scaffolds, concentrating the micrococcal nuclease (MNase) enzyme at the degradation interface to achieve localized and efficient DNA cleavage. This sequential mechanism disrupted the self-perpetuating cycle of ROS amplification and NET-driven inflammation, thereby alleviating systemic inflammatory dysregulation. Functionally, MOF-G3-MNase treatment led to comprehensive mitigation of sepsis pathology. In murine models, it markedly decreased circulating dsDNA and CitH3, suppressed cytokine storms (IL-1β, IL-6 and TNF-α), alleviated multi-organ injury, decreased the NADP⁺/NADPH ratio and attenuated GSDMD cleavage-associated activation, and improved survival—superior to all single-component controls. Importantly, ex vivo validation with patient-derived neutrophils and plasma demonstrated comparable outcomes, supporting the translational potential and clinical relevance of the nanoplatform. The reduction of ROS fluorescence intensity, normalization of nuclear morphology, and reduction in signal in patient samples collectively supported effective inhibition of NETosis and efficient NET degradation under clinically relevant conditions. The comparison with DNase I and GSK484 further supports the advantage of the integrated MOF-G3-MNase design. DNase I mainly promotes extracellular DNA degradation, whereas GSK484 inhibits PAD4-dependent NETosis. In contrast, MOF-G3-MNase combines NET capture, DNA scaffold degradation, and ROS modulation, which may explain its stronger reduction of circulating dsDNA and CitH3. These findings suggest that NET degradation and immunomodulation are functionally linked rather than completely independent processes. By capturing NETs through G3 and degrading the DNA scaffold through MNase, MOF-G3-MNase may reduces NET-associated DAMP exposure, while the MOF component attenuates ROS-driven inflammatory amplification. Although NETs and ROS contribute to antimicrobial host defense, excessive NET accumulation and oxidative stress can amplify systemic inflammation and organ injury during sepsis. Our blood CFU data showed that MOF-G3-MNase did not increase bacterial burden, but instead reduced blood CFU counts in CLP mice, suggesting that the nanocomposite mitigated pathological NET/ROS-driven inflammation while preserving systemic bacterial clearance. The comparison among MOF, MOF-G3, MOF-MNase, and MOF-G3-MNase also helped delineate the contribution of each functional module. MOF mainly contributed to ROS scavenging and inflammatory modulation, G3 enhanced NET association through electrostatic capture, and MNase mediated enzymatic degradation of the NET-DNA scaffold. The relatively limited effects of individual formulations indicate that targeting only one step is insufficient to fully suppress NET-associated inflammatory amplification. In contrast, MOF-G3-MNase integrates NET capture, DNA scaffold degradation, and ROS modulation, resulting in a more robust reduction of NET burden and inflammatory cytokine release. Importantly, this integrated effect may help rebalance the dysregulated immune response in sepsis by limiting excessive NET-associated danger signals and ROS-driven cytokine amplification, rather than broadly suppressing host immune defense. Mechanistically, transcriptomic and bioinformatic analyses revealed that MOF-G3-MNase therapy triggered broad chromatin remodeling and transcriptomic reprogramming in septic neutrophils. RNA-seq identified 1,168 differentially expressed genes—predominantly down-regulated—indicative of widespread transcriptional silencing of pro-inflammatory and oxidative stress pathways, consistent with MNase-mediated chromatin remodeling. Up-regulated genes such as Hspa5 and Dnmt3l reflected enhanced stress tolerance and epigenetic stabilization, whereas down-regulated metabolic genes (Slc26a1, Aldh3b2) signaled suppression of aberrant energy flux under septic stress. KEGG analysis highlighted significant enrichment in sulfur metabolism, ferroptosis, and porphyrin pathways, linking nanocatalytic redox regulation to restored cellular homeostasis. GO terms such as “response to stimulus” and “oxidoreductase activity” further corroborated the observed antioxidant and tissue-protective phenotypes. These transcriptomic insights provide molecular evidence that MOF-G3-MNase not only suppresses NETosis phenotypically but also is associated with transcriptional reprogramming toward a less inflammatory state (*42*). Although MOF-G3-MNase markedly reduced inflammatory cytokine protein levels, RNA-seq did not show uniform downregulation of all inflammatory cytokine-related transcripts. Such transcript–protein discrepancies are expected because mRNA abundance does not always predict protein abundance or pathway activity, owing to post-transcriptional regulation, differential RNA/protein half-lives, translational control, protein turnover, and post-translational modification. Therefore, the RNA-seq data were interpreted as evidence of transcriptional remodeling, whereas the reduction in inflammatory output was supported by ELISA measurement of TNF-α and IL-6 and by Western blot analysis showing reduced p-NF-κB p65 phosphorylation. The reduction in NET burden was further supported by decreased dsDNA, CitH3, and direct NET imaging analyses, while reduced GSDMD-N levels indicated attenuation of GSDMD cleavage-associated inflammatory activation. Compared with previous single-target strategies—such as ROS scavengers, DNase therapy, or immunosuppressants—MOF-G3-MNase achieves spatiotemporal precision and mechanistic complementarity, combining catalytic antioxidant protection with direct NETs degradation. This synergy enables simultaneous control of upstream triggers and downstream consequences of NETosis, preventing the feedback amplification that underlies cytokine storm and organ dysfunction. Moreover, the intrinsic stability and modularity of the MOF scaffold may provide a versatile platform adaptable to other enzymes or biomolecules, suggesting broad utility for NET-associated pathologies such as acute lung injury, autoimmune vasculitis, and thrombosis. While these findings reveal substantial therapeutic promise, several limitations remain. The CLP model, though widely accepted, does not fully recapitulate the heterogeneity of clinical sepsis. Although the present study demonstrates promising therapeutic efficacy and favorable short-term biocompatibility, the long-term biosafety of metal-containing MOF materials remains an important consideration. Because MOF-G3-MNase contains Cu and Zr, potential metal ion release, organ accumulation, delayed clearance, and chronic tissue toxicity should be carefully evaluated. To partially address this issue, we performed a 30-day biosafety assessment in healthy mice. Plasma biochemical markers, including ALT, AST, CREA, and BUN, showed no significant differences between the control and MOF-G3-MNase-treated groups, and H&E staining of major organs revealed no obvious histopathological abnormalities. These results suggest that MOF-G3-MNase exhibits acceptable 30-day biosafety under the current experimental conditions. Nevertheless, further long-term studies are still required to fully define its safety profile, including quantitative ICP-MS analysis of Cu and Zr biodistribution and clearance, hematological evaluation, and assessment of chronic inflammatory or immune responses. The relative contribution of individual components (MOF818, PAMAM-G3, MNase) to overall efficacy also warrants systematic dissection through gene-knockout or inhibitor-based models. Future studies should expand toward scalable synthesis, pharmacological optimization, and combination regimens with antibiotics or immune modulators to facilitate clinical translation. In conclusion, our findings establish MOF-G3-MNase as a prototype of cascade-responsive nanomedicine that orchestrates redox regulation, NET capture and degradation, and immune recalibration to combat sepsis. By bridging catalytic nanochemistry with immunopathology, this study introduces a generalizable paradigm for the design of multifunctional nanotherapeutics capable of restoring immune homeostasis in NET-driven inflammatory diseases. We have developed a multifunctional nanocomposite material MOF-G3-MNase that synergistically acts on the accumulation of ROS and the dysregulation of NETs in sepsis. By combining the antioxidant MOF818 with NET-capturing PAMAM-G3 and NET-degrading MNase, the system achieved ROS clearance, reduced oxidative stress, and decreased the NADP⁺/NADPH ratio. NETs clearance: Inhibit the degradation of existing NETs and suppress further NETosis through GSDMD. Treatment also reduced pro-inflammatory cytokine levels and alleviated multiorgan injury. MOF-G3-MNase improved survival in the murine sepsis model, while experiments using patient-derived neutrophils and plasma supported its clinical relevance. These results highlight the potential of multitarget nano therapy in treating sepsis and other inflammatory diseases driven by NETs. Our research not only provides a new material solution, but also promotes the understanding of the pathophysiology of sepsis, paving the way for future translational applications.

## MATERIALS AND METHODS

### Synthesis of MOF818 nanocatalysts

Cu(NO_3_)_2_·3H_2_O (124 mg), ZrOCl_2_·8H_2_O (42.5 mg), 1H-pyrazole-4-carboxylic acid (H_2_PyC) (32.5 mg), and trifluoroacetic acid (120 μL) were dissolved in 20 mL of N,N-dimethylformamide (DMF) and evenly dispersed by ultrasonication. The mixture was then transferred into a 50 mL polytetrafluoroethylene-lined autoclave and heated at 100°C for 8 h. After naturally cooling to room temperature, the resulting blue crystals were collected.

### Synthesis of MOF-G3-MNase nanocatalysts

MOF818 synthesized in the previous step was dispersed in deionized water. Briefly, 20 mg of MOF818 was dispersed in 1 mL of deionized water and activated with EDC (63.3 mg; molecular weight, 191.70) and NHS (38.0 mg; molecular weight, 115.1). PAMAM-G3-NH_2_ (50 mg) was then added to the activated MOF818 suspension, followed by washing three times with deionized water. The obtained MOF-G3 was redispersed in 1 mL of deionized water, incubated with 40 U of MNase, and washed three times to obtain MOF-G3-MNase.

### Characterization

A Tecnai G2 F30 electron microscope (FEI, Netherlands) was used to acquire transmission electron microscopy (TEM) images, energy-dispersive x-ray spectroscopy (EDX) elemental maps, and high-angle annular dark-field (HAADF) images. A Gemini SEM 300 microscope (Zeiss, Germany) was used to obtain scanning electron microscopy (SEM) images. A Zetasizer Nanoseries instrument (Malvern, UK) was used to measure hydrodynamic size and zeta potential. X-ray photoelectron spectroscopy (XPS) was performed using a K-Alpha system (Thermo Scientific). UV–vis spectra were acquired using a UV2700i spectrophotometer (Shimadzu, Japan). Fourier transform infrared spectroscopy was performed using a Nicolet iS19 spectrometer (Thermo Scientific). Inductively coupled plasma optical emission spectroscopy (ICP-OES) was used to measure copper and zirconium ion concentrations in the MOF nanocatalysts.

### ROS scavenging/SOD-like activity assay

The SOD-like activity of MOF-G3-MNase was evaluated using a total superoxide dismutase (SOD) activity assay kit based on the nitroblue tetrazolium (NBT) reduction method according to the manufacturer’s instructions. Briefly, the test sample was mixed with SOD assay buffer, NBT/enzyme working solution, and reaction initiator working solution, and the absorbance at 550 nm was continuously monitored for 10 min.

The superoxide anion scavenging efficiency was calculated according to the inhibition of NBT reduction. Electron paramagnetic resonance (EPR) spectroscopy was further used to evaluate the superoxide scavenging activity of MOF-G3-MNase. In brief, MOF-G3-MNase was added into Tris-HCl buffer (0.1 M, pH 7.2) containing xanthine (X) and 5,5-dimethyl-1-pyrroline N-oxide (DMPO). The final concentrations of MOF-G3-MNase, X, and DMPO were 10 μg mL^−1^, 0.4 mM, and 100 mM, respectively. Xanthine oxidase (XO, 0.125 U/mL) was then added to initiate superoxide generation, and the EPR spectra were subsequently recorded.

### Animals and treatments

The animal study protocol was approved by the Tongji University Animal Ethics Committee (TJBH08425107). A mouse model of sepsis was established using the cecal ligation and puncture (CLP) technique. Eight-week-old male C57BL/6 mice weighing 20–25 g were selected, fasted for 12 h before surgery with free access to water, and randomly divided into the CLP group and the sham surgery group. Mice were anesthetized with isoflurane inhalation anesthesia (5% for induction and 2% for maintenance). After the disappearance of the pain reflex was confirmed, mice were fixed on a sterile operating table. The abdominal hair was shaved, and the surgical area was disinfected sequentially with iodine and 75% ethanol. A 1 cm incision was made along the abdominal midline, the peritoneum was gently separated, and the cecum and adjacent intestine were exposed. The distal half of the cecum was ligated with 4–0 silk thread while avoiding damage to the mesenteric vessels, ensuring partial retention of intestinal contents without complete obstruction. The distal cecal wall was punctured once with a 21G needle, and gentle pressure was applied to the cecum to allow a small amount of feces to leak into the abdominal cavity. The cecum was then returned to the abdominal cavity, and the peritoneum and skin were sutured layer by layer. The incision was cleaned with sterile saline. Pre-warmed saline was administered subcutaneously at 1 mL/100 g to compensate for fluid loss. Mice in the sham group underwent laparotomy, cecal exposure, and reduction without ligation or puncture. For the in vivo biosafety assessment, eight-week-old male C57BL/6 mice weighing 20–25 g were obtained from Spofu Corporation. MOF-G3-MNase was administered intraperitoneally at a dose of 20 mg/kg twice. This dosing schedule was designed with reference to a previous report (*43*) using a prophylactic/perioperative treatment strategy in experimental sepsis, aiming to evaluate whether early intervention could attenuate the initial inflammatory cascade after CLP. ALT, AST, BUN, and CREA levels were measured in the control and MOF-G3-MNase groups. H&E-stained sections of the heart, liver, spleen, lung, and kidney were also observed and compared between the two groups. DNase I and GSK484 were used as reference treatments. DNase I was administered (10 mg/kg i.p., samples collected at 24 h), and GSK484 was administered (10 mg/kg i.p., samples collected at 24 h) (*42*, *44*). Plasma samples were collected at 24 h after CLP for dsDNA and CitH3 measurement. For in vivo therapeutic studies, MOF818, MOF-G3, MOF-MNase and MOF-G3-MNase were administered at a dose of 20 mg/kg by intraperitoneal injection. In the CLP model, mice received the nanocomposites at 12 h before and 1 h after CLP surgery unless otherwise indicated. The same dose (20 mg/kg) was also used in the in vivo biosafety studies. All animal experiments comply with ARRIVE 2.0 guidelines.

### Patients

The sample size for the ex vivo validation using patient-derived plasma and neutrophils was estimated using G*Power software and further determined according to the availability of eligible clinical specimens. A total of 54 sepsis patients and 54 healthy controls were included. Fifty-four sepsis patients admitted to the ICU of Tongji University Affiliated Shanghai Fourth People’s Hospital, China, from July 2025 to December 2025 were recruited. Participants were excluded if they had clinical conditions or incomplete data that precluded reliable evaluation of the study outcomes, or if the investigators considered participation inappropriate because of concerns regarding participant safety or data validity. Samples were processed within 2 h after collection. Fifty-four healthy volunteers were enrolled as controls. This study was approved by the Institutional Medical Ethics Committee of Tongji University Affiliated Shanghai Fourth People’s Hospital (2025032–001), and informed consent was obtained from patient representatives. Baseline clinical characteristics, including age, sex, SOFA score, APACHE II score, pathogen type, and clinical outcome, were collected from the medical records of enrolled participants and summarized in table S1. Research involving human peripheral blood samples follows ICMJE recommendations.

### Neutrophil isolation

Human neutrophils (PMNs) and mouse bone marrow neutrophils were used in this study. Human blood samples were processed by plasma separation and centrifugation. Blood cells were diluted with an equal volume of PBS and layered onto Histopaque 1119 (Sigma) and Ficoll-Paque (Cytiva, USA) at a volume ratio of 2:1:1. After density gradient centrifugation, the PMN layer was collected. Red blood cells were lysed using red blood cell lysis buffer (BD Pharmingen, USA), and the cells were washed. Mouse bone marrow neutrophils were isolated using a Bone Marrow Neutrophil Isolation Kit (TBD Sciences, China). The tibia and femur were separated, and bone marrow was flushed and collected. The bones were rinsed several times until they turned white. The resulting cell suspension was subjected to density gradient centrifugation according to the manufacturer’s instructions. The bone marrow neutrophil layer was collected, washed, and lysed when necessary. The cells were centrifuged and resuspended for subsequent flow cytometry analysis.

### Flow cytometry analysis

PMNs were incubated with anti-CD15 antibody (BioLegend, USA), and mouse bone marrow neutrophils were incubated with anti-Ly6G (BioLegend, USA) and anti-CD11b antibodies (BioLegend, USA) to assess neutrophil purity. Data were collected using a BD FACS flow cytometer (figs. S1 and S2).

### Immunofluorescence staining

Isolated neutrophils were seeded at a concentration of 5 × 10^5^ cells/mL into 24-well cell culture plates preloaded with poly-L-lysine-coated coverslips and cultured for 2 h. After incubation, the culture medium was discarded, and the cells were washed three times with 1× PBS for 3 min each. Cells were fixed with 200 μL of 4% paraformaldehyde per well for 15 min. After fixation, the cells were washed three times with 1× PBS for 3 min each. Immunostaining blocking solution containing permeabilizing reagent was added to each well, and the cells were blocked at room temperature for 1 h. After blocking, 150 μL of primary antibody solution was added to each well and incubated overnight at 4°C. The next day, the primary antibody was removed, and the cells were washed three times with 1× PBS for 3 min each. Then, 150 μL of the corresponding fluorescent secondary antibody was added to each well and incubated at room temperature in the dark for 1 h. After incubation, the secondary antibody was discarded, and the cells were washed three times with 1× PBS. After incubation, the secondary antibody was discarded, and the cells were washed three times with 1× PBS. Diluted DAPI staining solution (1 μg/mL, 150 μL/well) was added and incubated in the dark for 10 min. After DAPI staining, the cells were washed three times with 1× PBS for 3 min each. The coverslips were removed, inverted onto glass slides, sealed with antifade mounting medium, and observed under a fluorescence microscope.

### ELISA analysis

Tumor necrosis factor-α (TNF-α), interleukin-6 (IL-6), and interleukin-1β (IL-1β) levels in mouse plasma were detected using ELISA kits from Elabscience (China). TNF-α, IL-6, and IL-1β levels in PMN supernatants were measured using human ELISA kits from Elabscience (China). All experiments were performed according to the manufacturer’s instructions.

### Detection of circulating NETs

Circulating NET markers, including double-stranded DNA (dsDNA) and citrullinated histone H3 (CitH3), were detected in plasma and cell supernatants using a CitH3 ELISA kit (Cayman Chemical, USA) and a PicoGreen dsDNA quantitation kit (Solarbio, China). All experiments were performed according to the manufacturers’ instructions.

### Western blot analysis

Neutrophils were isolated from mouse bone marrow at 24 h post-CLP and lysed in RIPA lysis buffer on ice for 15 min to extract proteins. The cell lysates were centrifuged at 12,000g for 15 min at 4°C, and the supernatants were collected. Protein concentrations were measured using a BCA protein assay kit (Beyotime, China). Proteins were separated by SDS-PAGE and transferred onto PVDF membranes. After blocking, the membranes were incubated with primary antibodies overnight at 4°C. PMN and mouse bone marrow neutrophil proteins were incubated with anti-citrullinated H3 antibody (1:1000; ab5103; Abcam, UK). Anti-β-actin was used as the internal control.

### Detection of intracellular ROS levels

Intracellular ROS levels were measured using a reactive oxygen species assay kit (Beyotime, China). Briefly, neutrophils seeded in 24-well culture plates were washed with PBS and incubated with 200 μL of DCFH-DA working solution at 37°C for 30 min. Fluorescence images were acquired using an Olympus microscope, and DCFH-DA fluorescence intensity was measured.

### Measurement of the NADP⁺/NADPH ratio

The NADP⁺/NADPH ratio in PMNs was measured using a WST-8–based NADP⁺/NADPH assay kit (Beyotime, China). According to the manufacturer’s instructions, each PMN sample was divided into two equal portions. One aliquot containing NADP^+^ and NADPH was kept on ice for 15 min, whereas the other aliquot was incubated at 60°C for 30 min to deplete NADP^+^ and retain NADPH. After centrifugation, 100 μL of G6PDH working solution was added to each sample well and incubated at 37°C in the dark for 10 min. The optical density at 450 nm was measured using a microplate reader, and the NADP^+^/NADPH ratio was calculated.

### Histopathological and clinical scoring

Histopathological injury was evaluated using H&E-stained tissue sections by investigators blinded to the experimental groups. For each tissue, representative fields were examined and scored according to the severity of pathological changes. Lung injury was assessed based on inflammatory cell infiltration, alveolar wall thickening, alveolar structural disruption, hemorrhage, and edema. Clinical scores were evaluated after CLP surgery by investigators blinded to the treatment groups. The scoring criteria included general appearance, spontaneous activity, response to external stimulation, posture, piloerection, and respiratory status. Higher scores indicated more severe clinical manifestations of sepsis.

### Bacterial burden assessment by CFU counting

Blood bacterial burden was assessed by CFU counting. Briefly, blood samples were collected from mice at 24 h after CLP surgery, serially diluted in sterile PBS, and plated onto agar plates. After incubation at 37°C, bacterial colonies were counted and expressed as CFU/mL of blood.

### RNA-seq sample preparation and experimental design

For RNA-seq analysis, neutrophils were isolated from mice at 24 h after CLP. The experimental design included the CLP and MOF-G3-MNase groups, with six biological replicates per group. Total RNA was extracted from isolated neutrophils and subjected to library construction and high-throughput RNA sequencing. Differential expression analysis was performed to identify sepsis-induced transcriptional alterations and MOF-G3-MNase-associated transcriptomic changes in neutrophils. All RNA-seq and proteomic data reporting adheres to MIBBI series standards (MIAME, MIAPE).

### Statistical analysis

Experimental data were statistically analyzed and plotted using GraphPad Prism 9 software. Measurement data were tested for normal distribution and are presented as mean ± SEM. Overall comparisons among multiple groups were performed using one-way ANOVA followed by Tukey’s post hoc test. Comparisons between two groups were performed using an unpaired t-test when appropriate. A value of *P* < 0.05 was considered statistically significant.
