# Supplementary material for: Sequential clearance of neutrophil extracellular traps for precision therapy of sepsis
Source: Sci Adv. 2026 Sep 2;12(36):eaef3930. doi: 10.1126/sciadv.aef3930 (PMC13537274; doi:10.1126/sciadv.aef3930)
Supplement: Supplementary file 1 [file sciadv.aef3930_sm.pdf]

Science Advances
AAAS

Supplementary Materials for

Sequential clearance of neutrophil extracellular traps for precision therapy of sepsis

Nan Wang *et al.*

Corresponding author: Qian Chen, chenqian1989@tongji.edu.cn; Cheng Li, chengli_2017@tongji.edu.cn

Sci. Adv. **12**, eaef3930 (2026)
DOI: 10.1126/sciadv.aef3930

This PDF file includes:

Figs. S1 to S21
Table S1

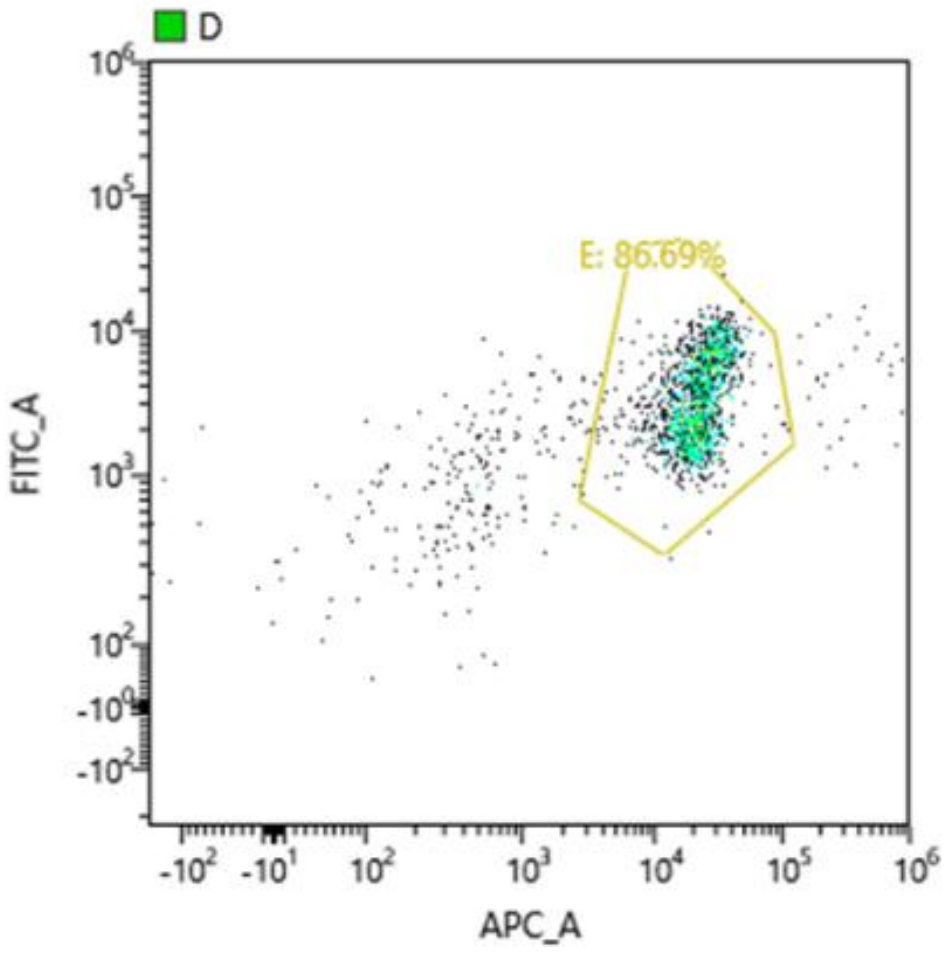

Figure S1. Using mice surface staining of neutrophil markers Ly6G and CD11b, and flow cytometry detection of BMDN purity;

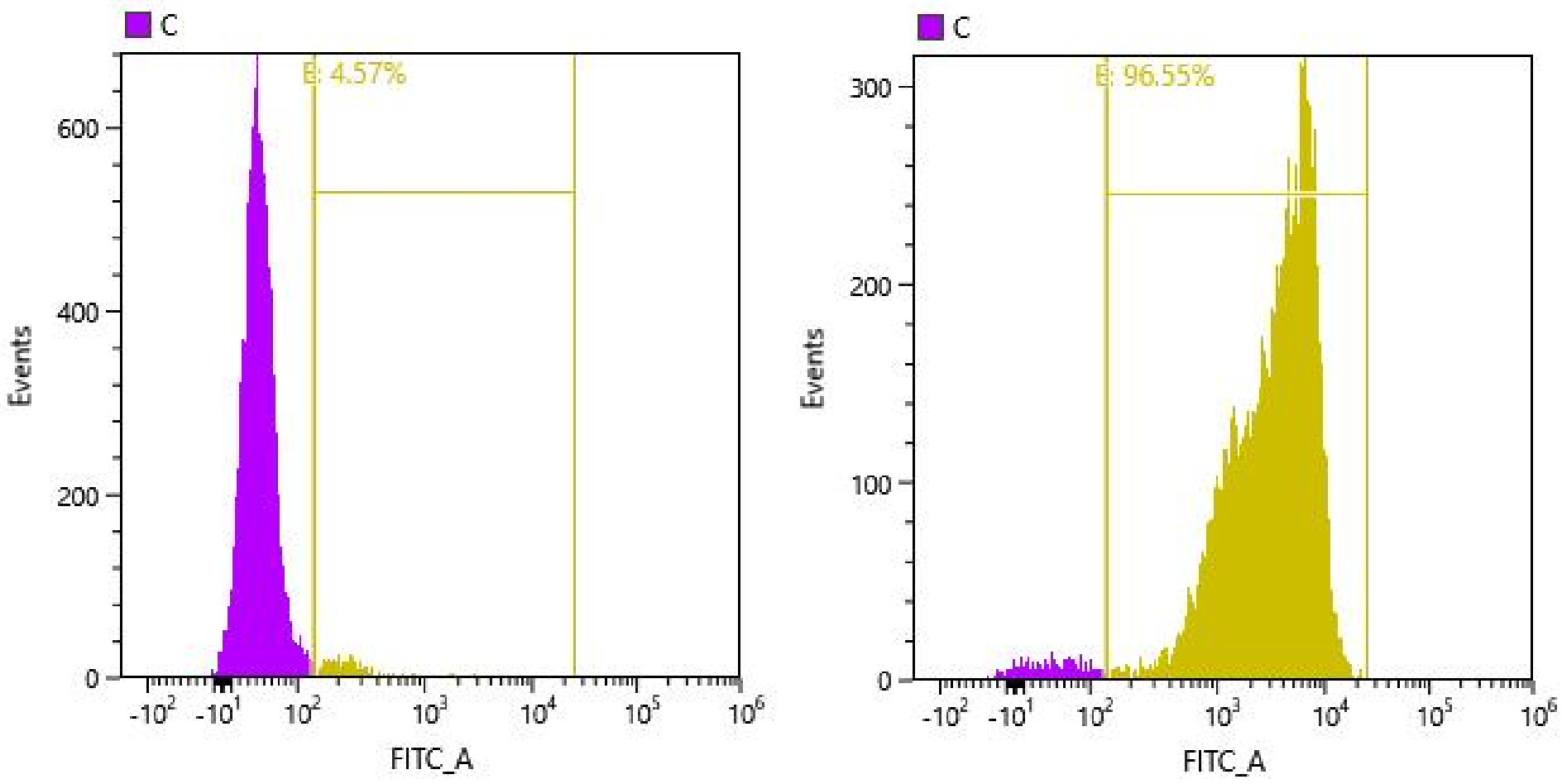

Figure S2. (B) Use neutrophil marker CD15 surface staining and flow cytometry to detect neutrophil purity.

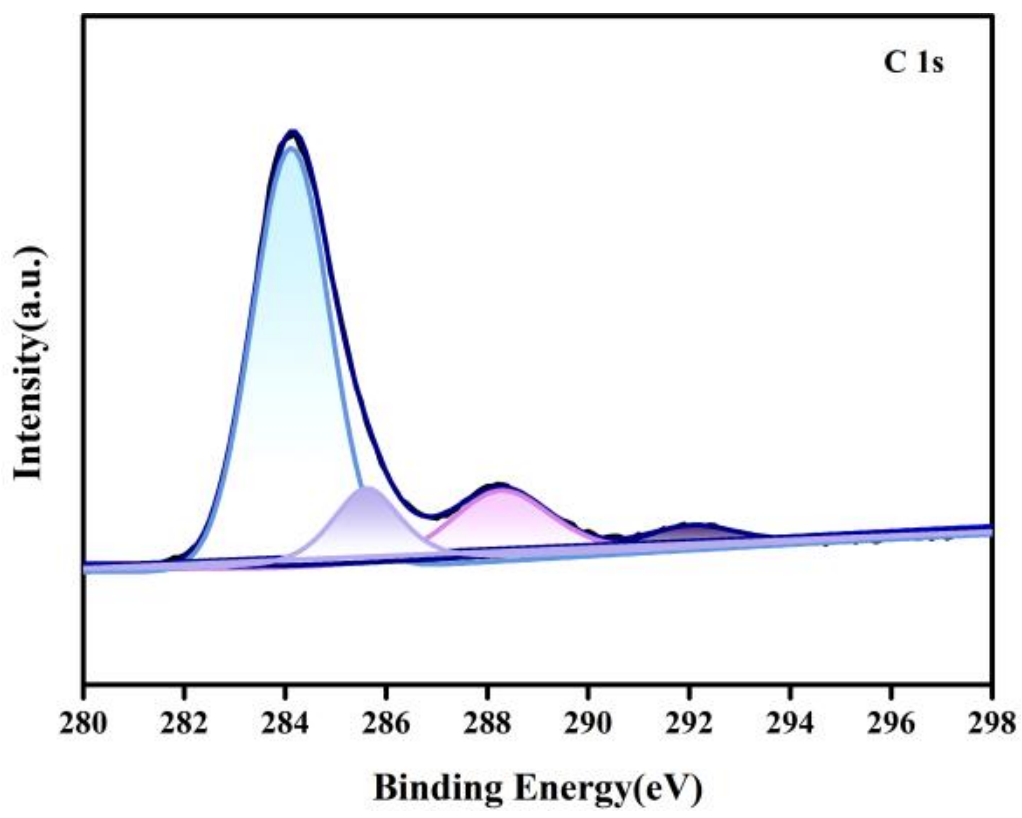

Figure S3 X-ray photoelectron spectroscopy (XPS): C spectrum.

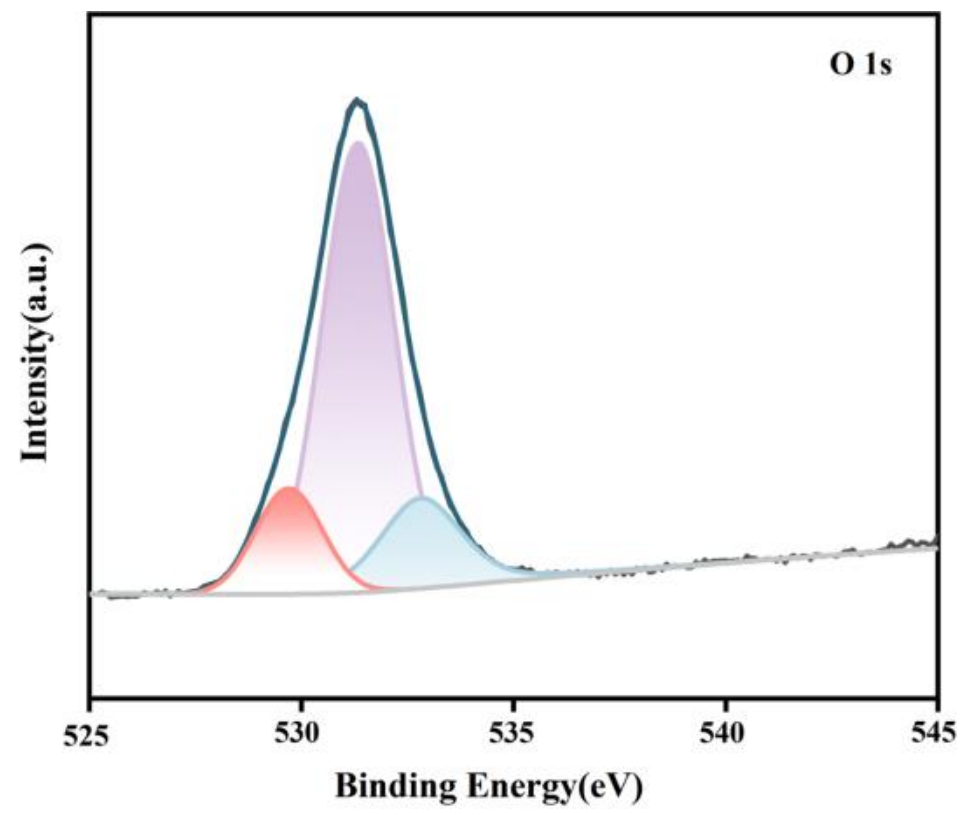

Figure S4 X-ray photoelectron spectroscopy (XPS): O spectrum.

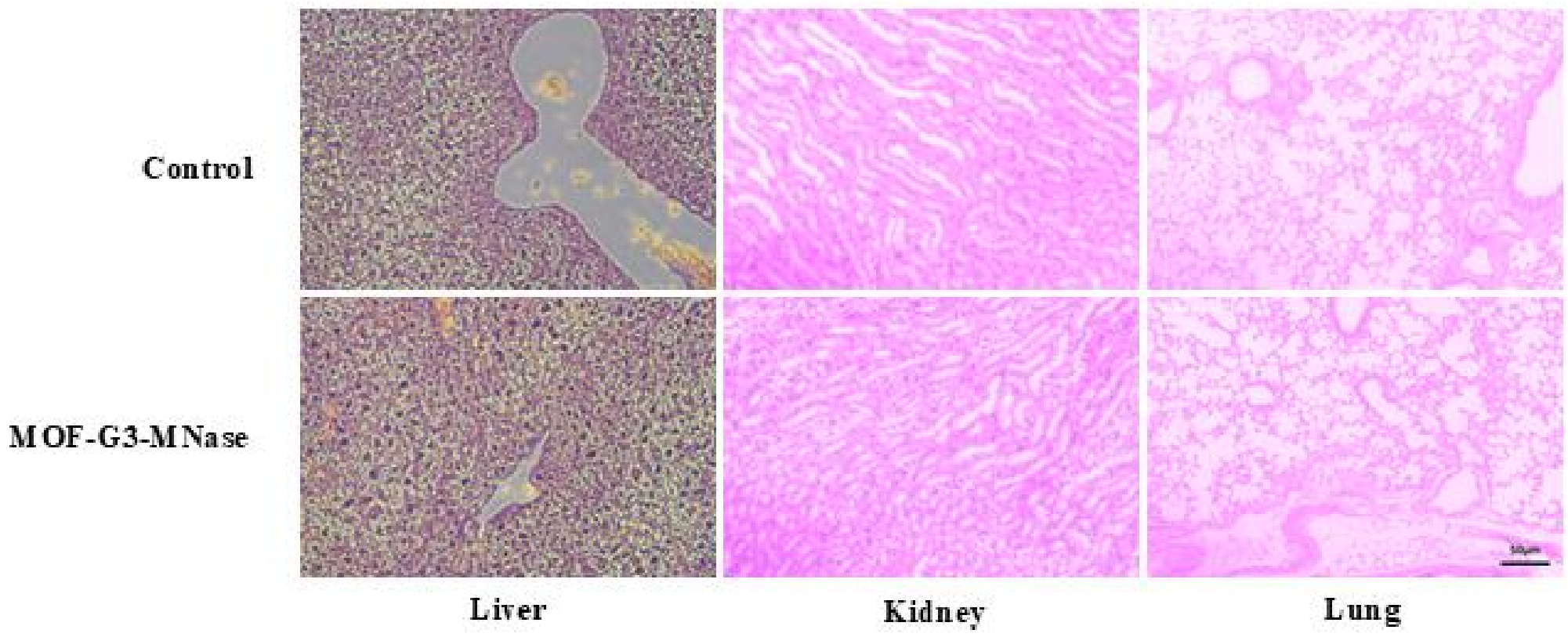

Figure S5 HE staining of liver, kidney, and lung tissue at 24 hours in the control group and MOF-G3-MNase injection group.

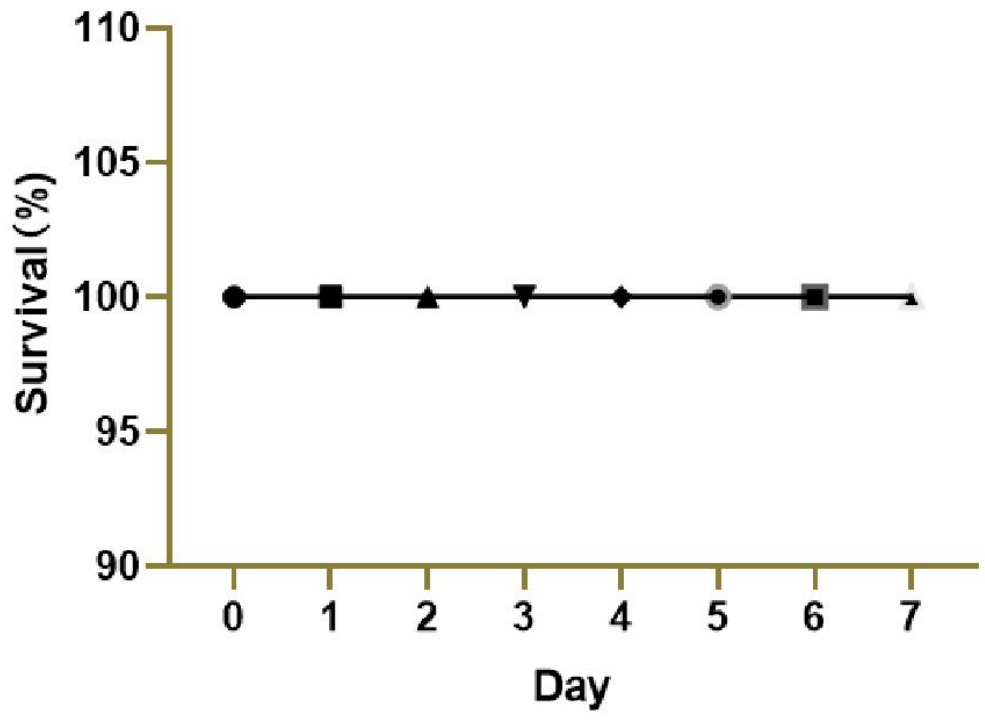

Figure S6 Survival rate of MOF-G3-MNase injection group mice.

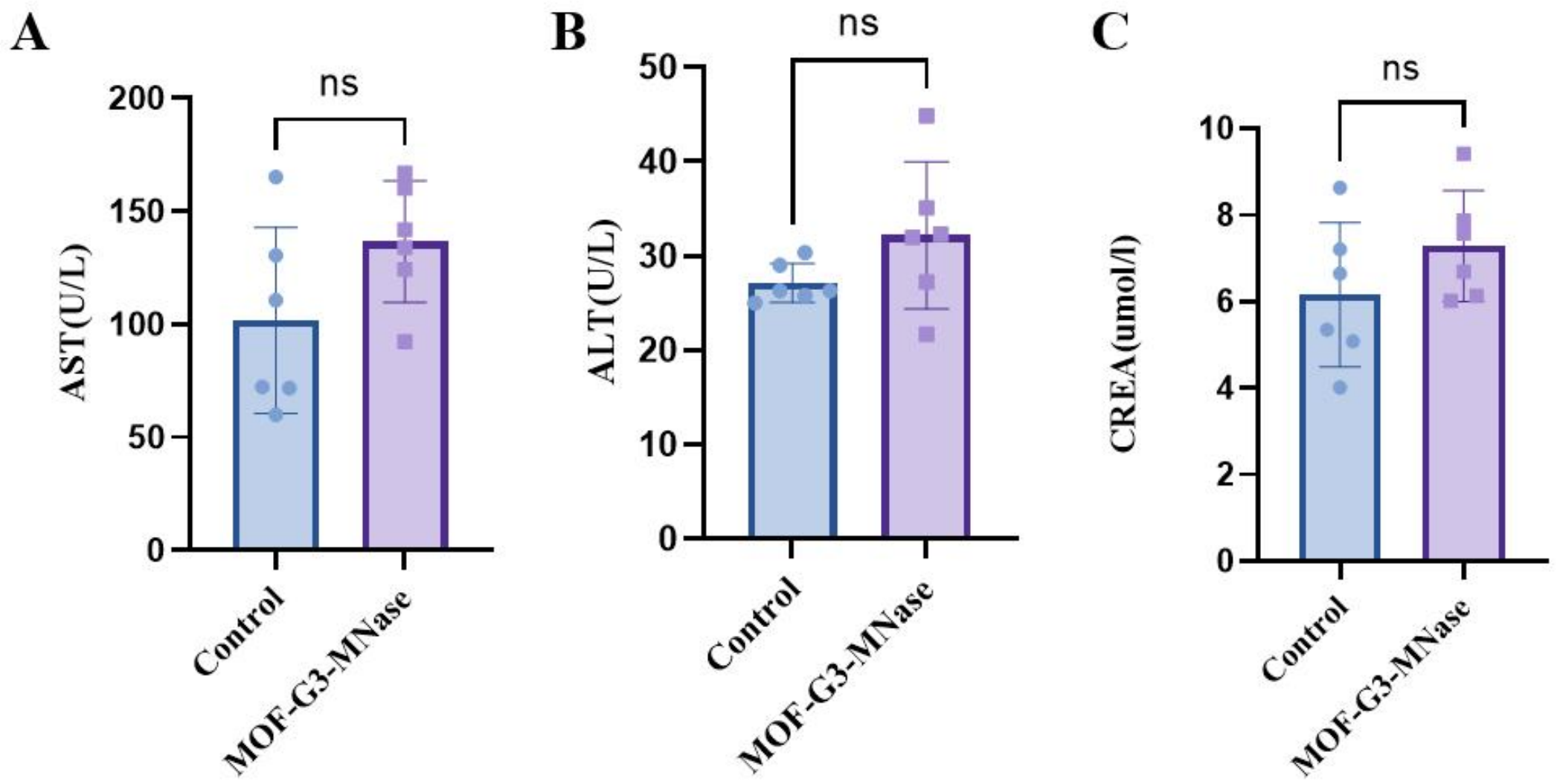

Figure S7 (A-C) The values of AST, ALT, and creatinine in the control group and MOF-G3-MNase injection group at 24 hours.

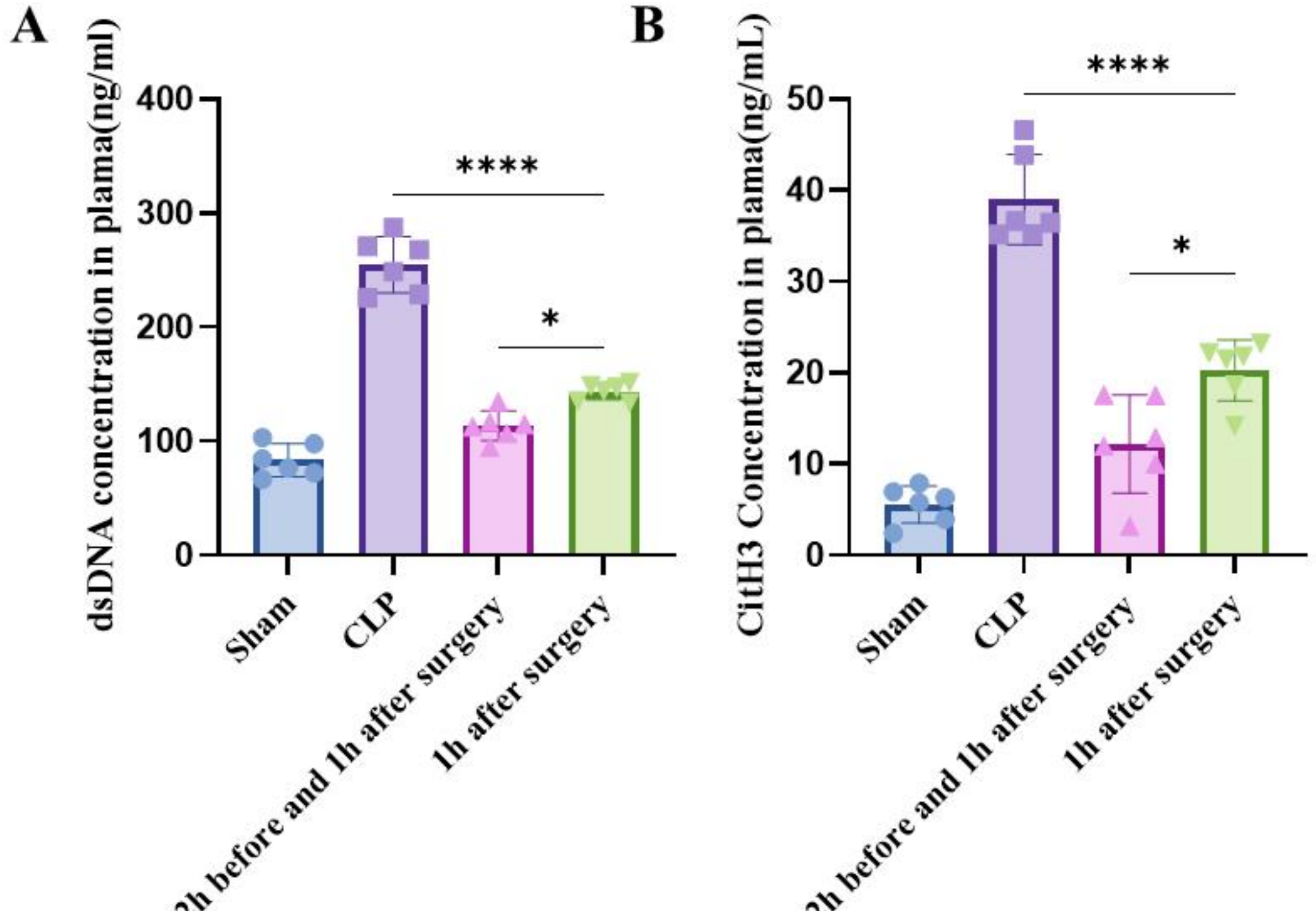

Figure S8 Therapeutic efficacy of post-treatment with MOF-G3-MNase in CLP-induced sepsis. To evaluate the therapeutic relevance of MOF-G3-MNase after sepsis onset, mice were subjected to CLP and treated with MOF-G3-MNase either at 12 h before and 1 h after CLP surgery or only at 1 h after CLP surgery. Sham and CLP mice received an equal volume of PBS. Blood samples were collected at 24 h after CLP for biochemical analyses. Clinical scores and survival were monitored

for 144 h after surgery. (A) Serum dsDNA levels were measured to evaluate circulating extracellular DNA/NET burden(n=6); (B) Serum CitH3 levels were measured as a NETosis-associated marker(n=6). Data are presented as mean ± SEM. Statistical analysis was performed using one-way ANOVA followed by Tukey's post hoc test. *P < 0.05, ****P < 0.0001; ns, not significant.

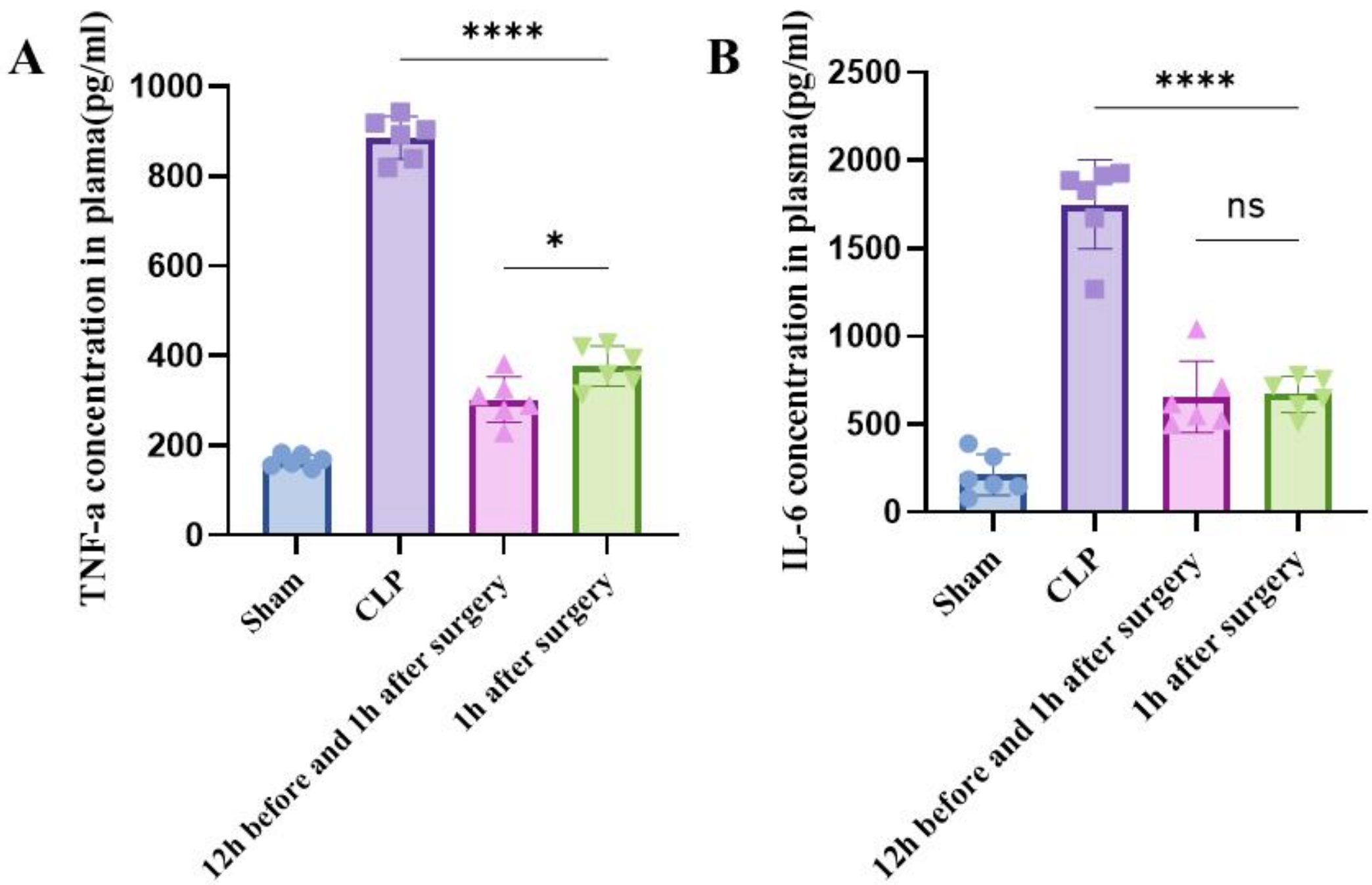

Figure S9 (A) Serum TNF-α levels were measured by ELISA(n=6); (B) Serum IL-6 levels were measured by ELISA (n=6). Data are presented as mean ± SEM. Statistical analysis was performed using one-way ANOVA followed by Tukey's post hoc test. *P < 0.05, ****P < 0.0001; ns, not significant.

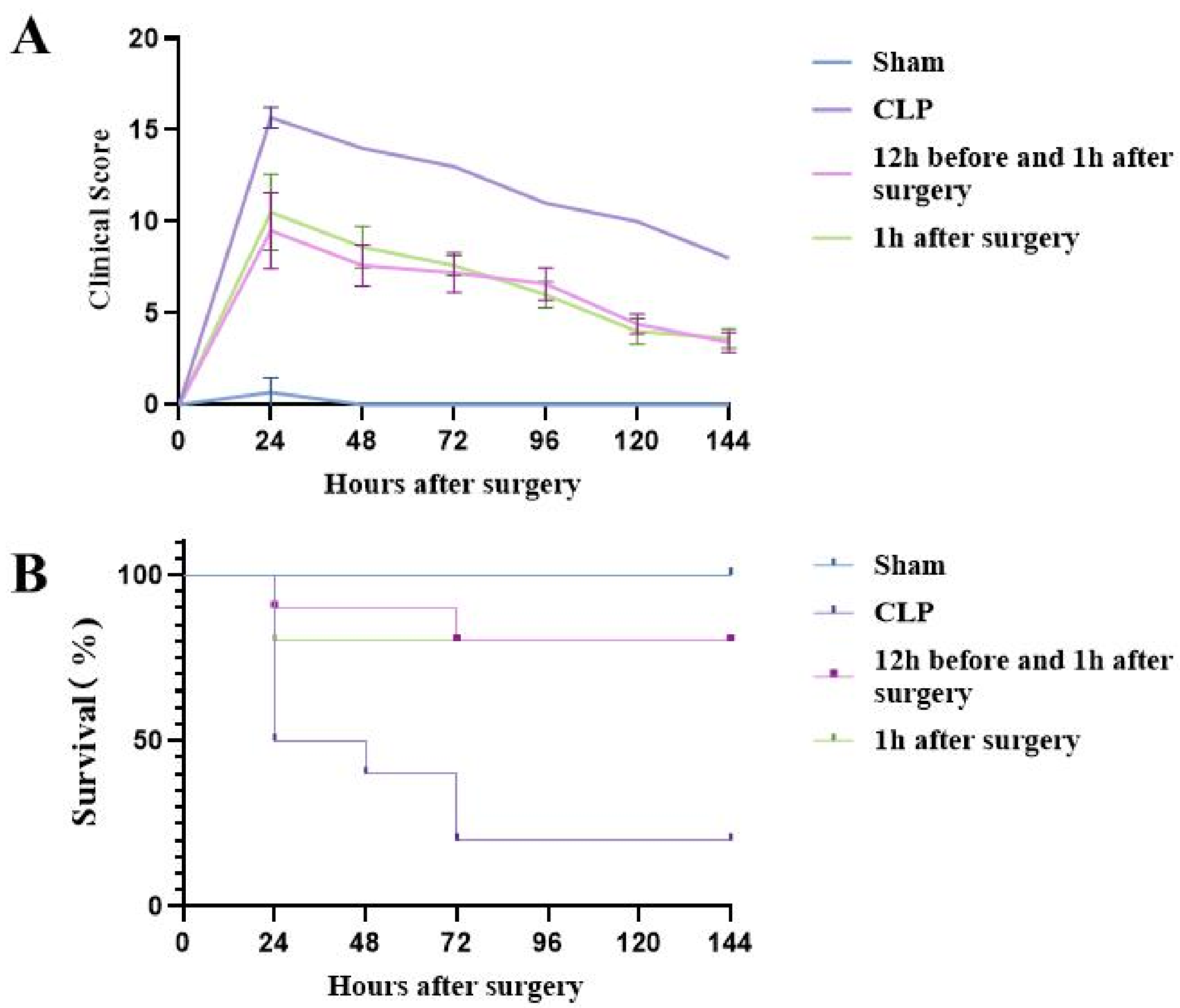

Figure S10 (A) Clinical scores of mice were recorded over 144 h after CLP(n=6); (B) Survival curves of mice were monitored for 144 h after CLP (n=10). Data are presented as mean ± SEM.

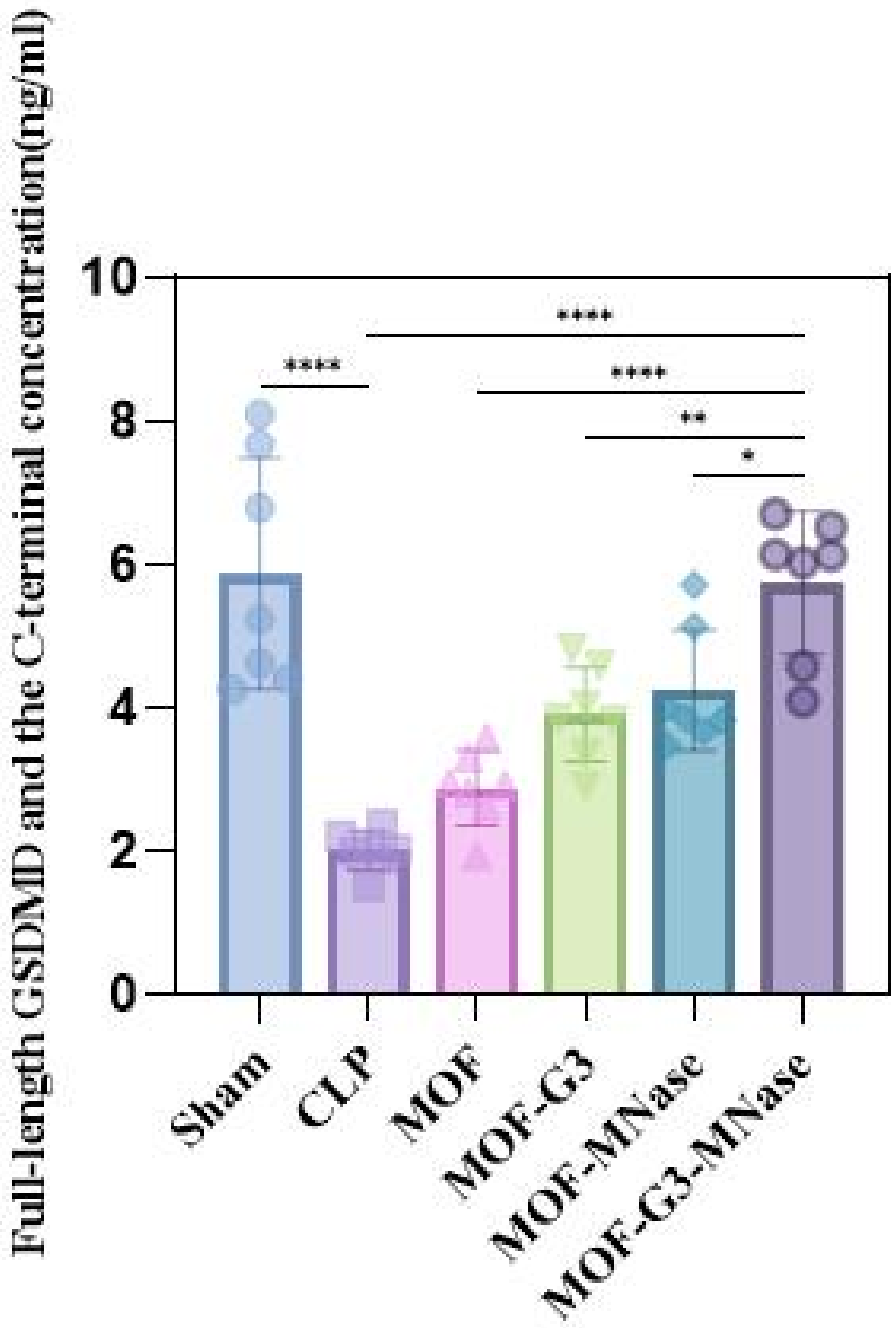

Figure S11 Determination of full-length GSDMD and the C-terminal levels in mice plasma by ELISA method (n=7 mice per group). Data are presented as mean ± SEM. Statistical analysis was performed using one-way ANOVA followed by Tukey's post hoc test. *P < 0.05, ****P < 0.0001; ns, not significant.

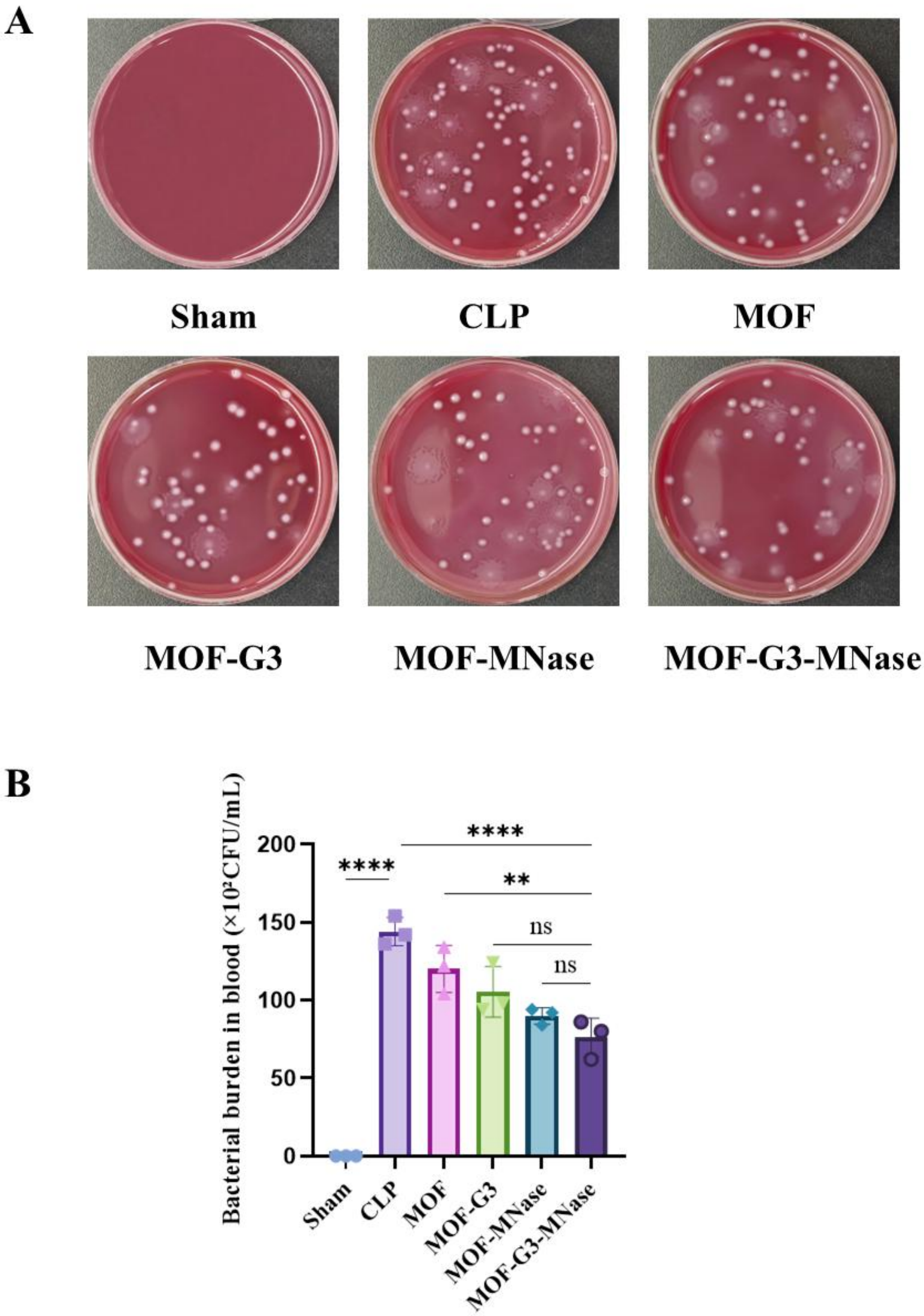

Figure S12. Effect of MOF-G3-MNase on blood bacterial burden in CLP-induced sepsis. (A) Representative images of bacterial colonies grown from blood samples collected from mice in different treatment groups at 24 h after CLP surgery. (B) Quantification of blood bacterial burden by CFU counting. CLP markedly increased blood bacterial burden compared with the Sham group, whereas MOF-G3-MNase treatment significantly reduced blood CFU counts compared with untreated CLP mice. These results indicate that MOF-G3-MNase did not compromise systemic bacterial clearance

while attenuating excessive NET/ROS-driven inflammatory responses. Data are presented as mean ± SD. Statistical significance was determined by one-way ANOVA followed by Tukey's multiple comparisons test. ns, not significant; **P < 0.01; ****P < 0.0001.

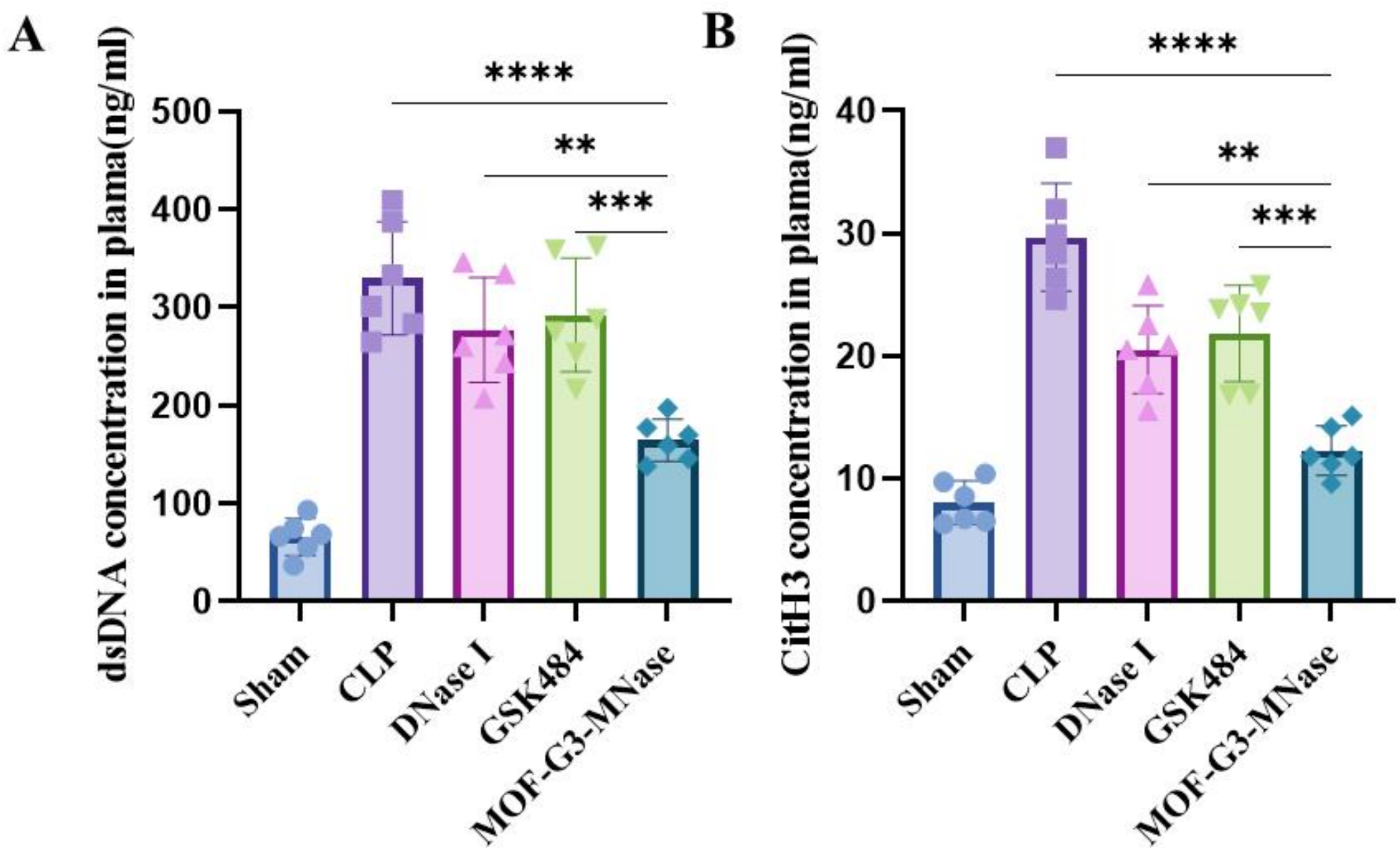

Figure S13. MOF-G3-MNase outperforms DNase I and GSK484 in attenuating NET-associated inflammation and improving survival in septic mice. (A, B) Quantification of plasma dsDNA and CitH3 levels, two representative NET-associated markers, in Sham, CLP, DNase I, GSK484, and MOF-G3-MNase groups at 24 h after CLP surgery. Data are shown as mean ± SD. Statistical significance was analyzed by one-way ANOVA followed by Tukey's post hoc test for ns, not significant; **P < 0.01; ***P < 0.001; ****P < 0.0001.

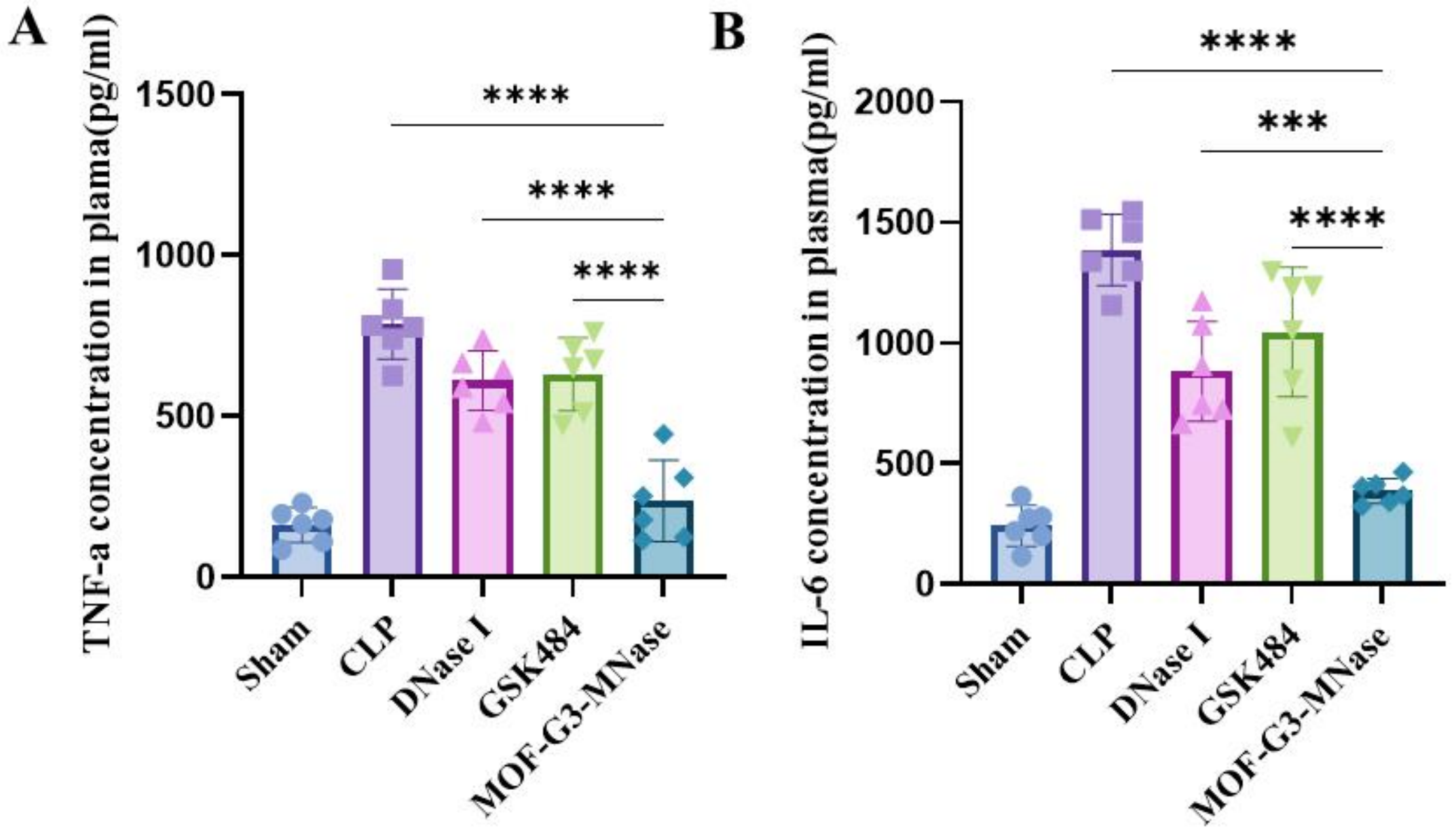

Figure S14 (A, B) Plasma TNF-α and IL-6 levels were measured to evaluate systemic inflammatory responses. DNase I-mediated NET degradation and GSK484-mediated PAD4 inhibition partially reduced NET-associated and inflammatory markers, whereas MOF-G3-MNase produced a more pronounced inhibitory effect. Data are shown as mean ± SD. Statistical significance was analyzed by one-way ANOVA followed by Tukey's post hoc test for ns, not significant; $^{**}P < 0.01$; $^{***}P < 0.001$; $^{****}P < 0.0001$.

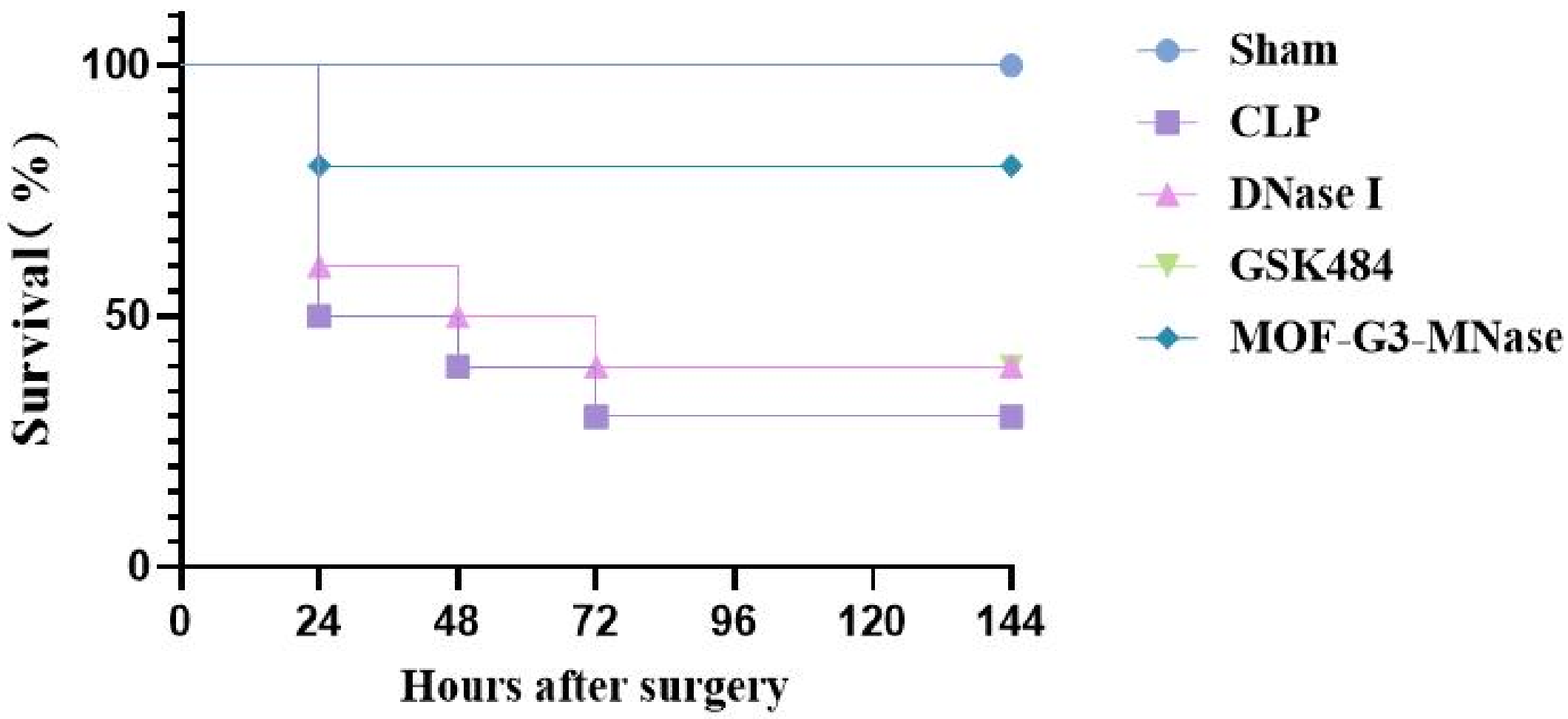

Figure S15 Kaplan–Meier survival analysis showing improved survival after MOF-G3-MNase

treatment. Data are shown as mean ± SD.

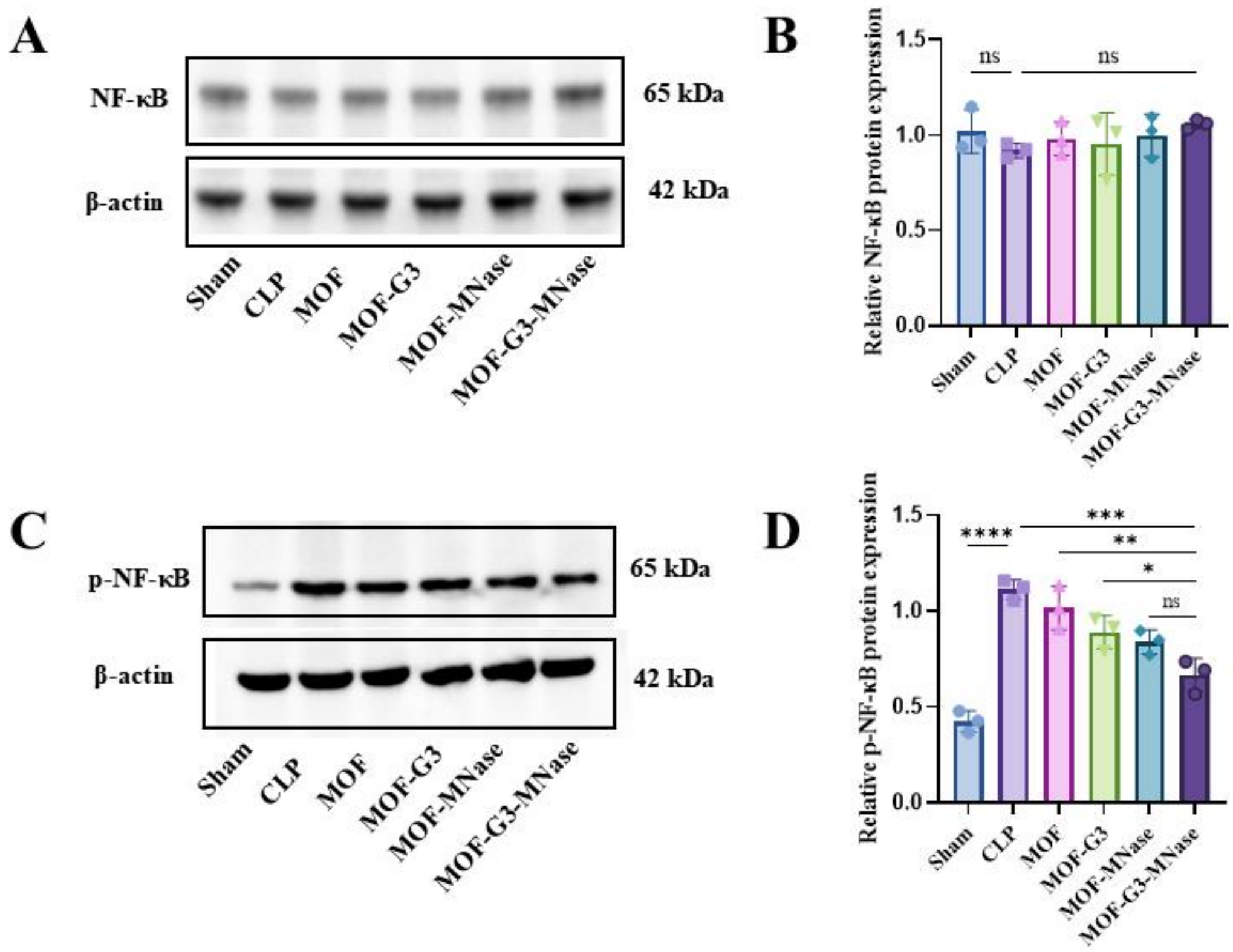

Figure S16. MOF-G3-MNase attenuates NF-κB activation in neutrophils after CLP-induced sepsis. (A, B) Representative Western blot images and quantitative analysis of total NF-κB p65 protein expression in neutrophils isolated from different treatment groups at 24 h after CLP surgery. Total NF-κB p65 expression showed no significant differences among groups. (C, D) Representative Western blot images and quantitative analysis of phosphorylated NF-κB p65 (p-NF-κB p65) expression. CLP markedly increased p-NF-κB p65 levels compared with the Sham group, whereas MOF-G3-MNase treatment significantly reduced p-NF-κB p65 expression. β-actin was used as the loading control. Data are presented as mean ± SD. Statistical significance was determined by one-way ANOVA followed by Tukey's multiple comparisons test. ns, not significant; *$P < 0.05$; **$P < 0.01$; ***$P < 0.001$; ****$P < 0.0001$.

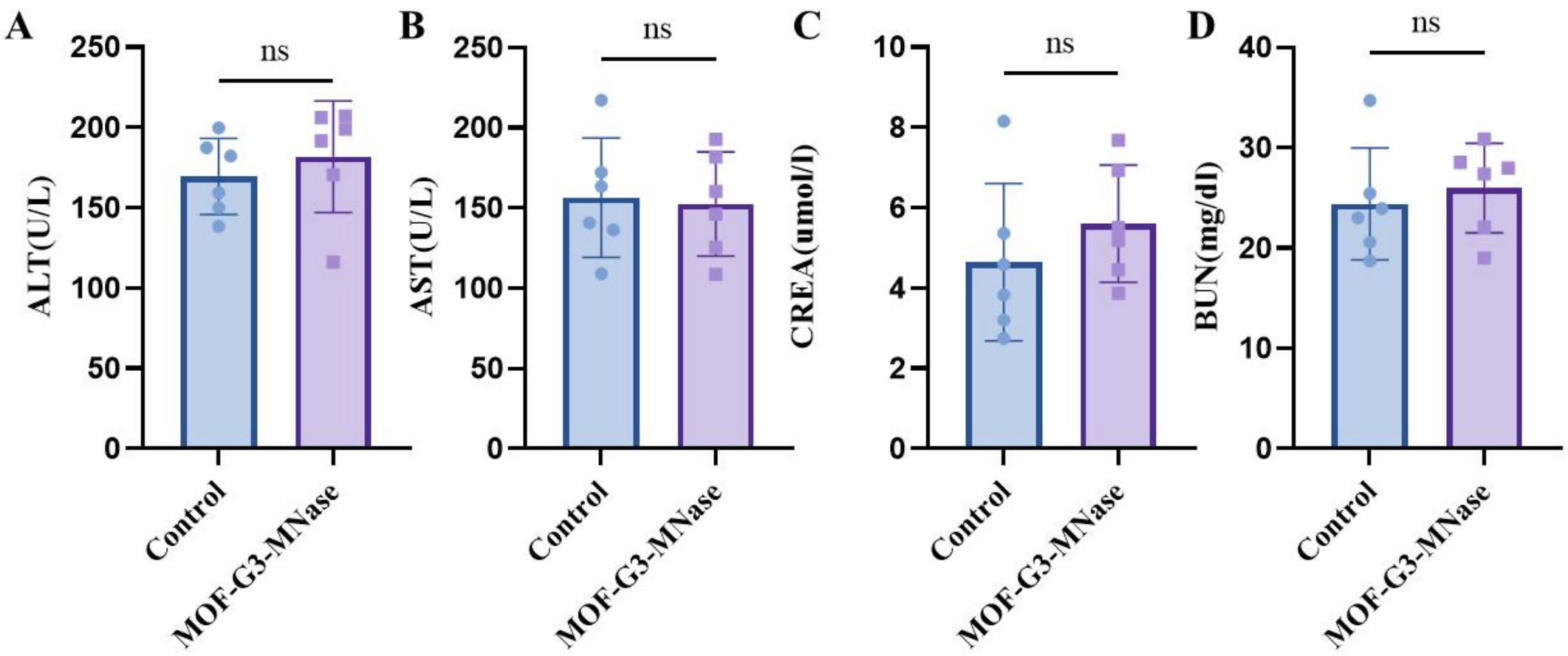

Figure S17 Thirty-day biosafety evaluation of MOF-G3-MNase in mice. (A–D) Serum biochemical analysis of mice at 30 days after MOF-G3-MNase administration, including alanine aminotransferase (ALT), aspartate aminotransferase (AST), creatinine (CREA), and blood urea nitrogen (BUN). No significant differences were observed between the control and MOF-G3-MNase groups. Data are presented as mean ± SD. Statistical significance was determined by unpaired two-tailed Student's t-test. ns, not significant.

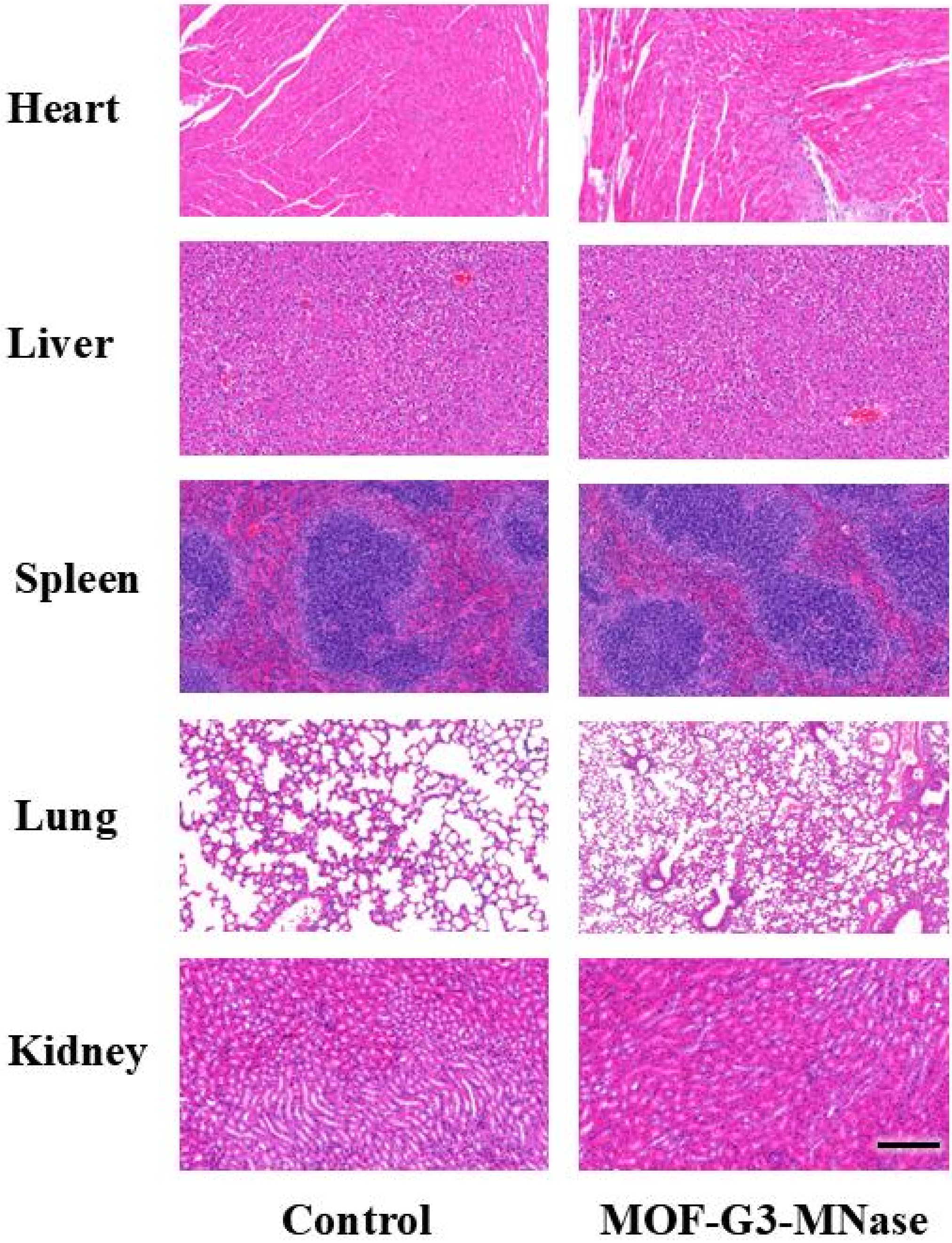

Figure S18 Representative H&E staining images of major organs, including heart, liver, spleen, lung, and kidney, collected at 30 days after treatment. No obvious histopathological abnormalities or tissue damage were observed in the MOF-G3-MNase-treated mice compared with the control group. Scale bar, 50 μm.

Characteristic	Sepsis patients
**Number of participants**	N=54
**Age, years**	71.3 ± 10.7
**Sex, male/female**	30/24
**SOFA score**	8.0 (6.25–10.0)
**APACHE II score**	21.5 (16.0–27.8)
**Pathogen type**	
Gram-negative bacteria	29(53.7%)
Gram-positive bacteria	13 (24.1%)
**Mixed infection**	1 (1.9%)
Culture-negative or not detected	11 (20.4%)
**28-day mortality**	7 (13.0%)

Table S1. Baseline characteristics of sepsis patients.

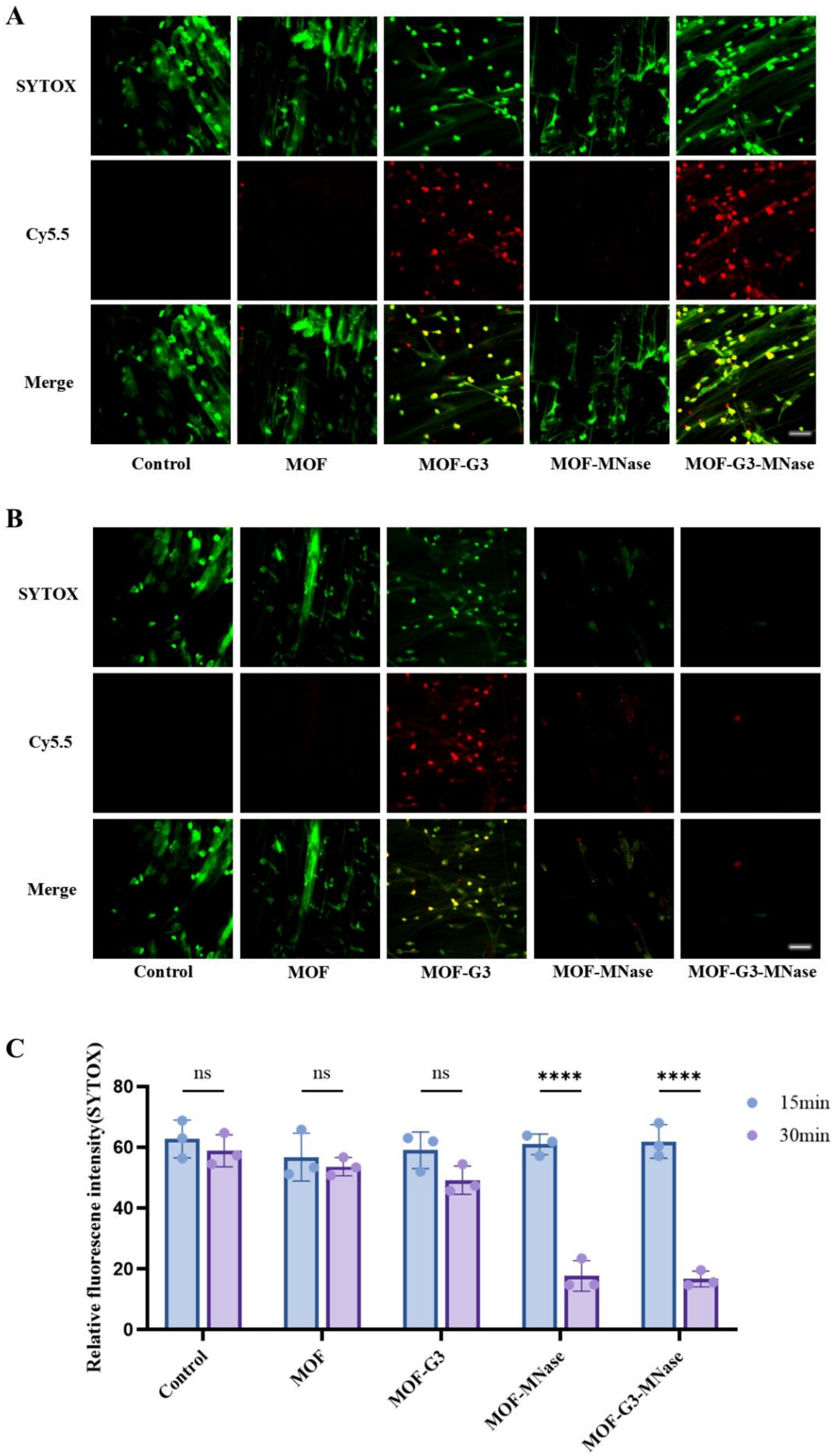

Figure S19 Time-course confocal imaging of MOF-G3-MNase-mediated NET capture and degradation. (A, B) Representative confocal images of pre-formed NETs incubated with Cy5.5-labeled nanoparticles

for 15 min (A) and 30 min (B). SYTOX Green was used to stain extracellular NET-DNA structures, and Cy5.5 fluorescence indicates nanoparticle localization. At 15 min, MOF-G3 and MOF-G3-MNase showed pronounced colocalization with SYTOX-positive NET structures, indicating rapid G3-mediated NET capture. At 30 min, the SYTOX-positive NET-DNA network was markedly reduced in the MOF-G3-MNase group, whereas NET structures were relatively preserved in the MOF-G3 group, supporting subsequent MNase-mediated degradation of the NET DNA backbone. Scale bar, 200 μm.

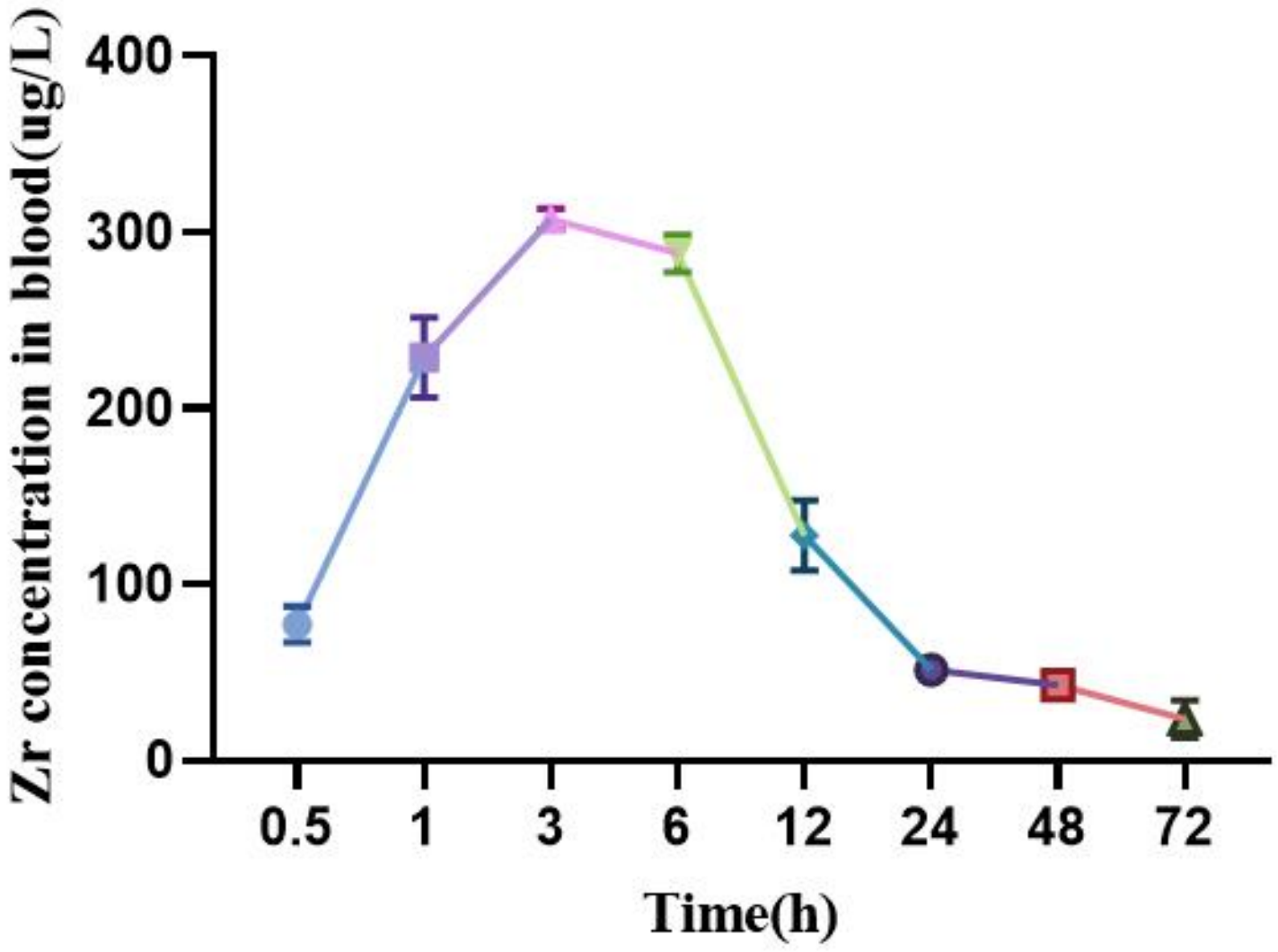

Figure S20. Blood pharmacokinetic profile of MOF-G3-MNase after intraperitoneal administration. Zr concentration in blood was quantified by ICP-MS at predetermined time points after intraperitoneal administration of MOF-G3-MNase. Zr was used as the elemental marker of the MOF framework. The blood Zr concentration increased after injection, reached a peak at approximately 3 h, and gradually decreased from 6 to 72 h, indicating systemic absorption followed by progressive blood clearance. Data are presented as mean ± SD.

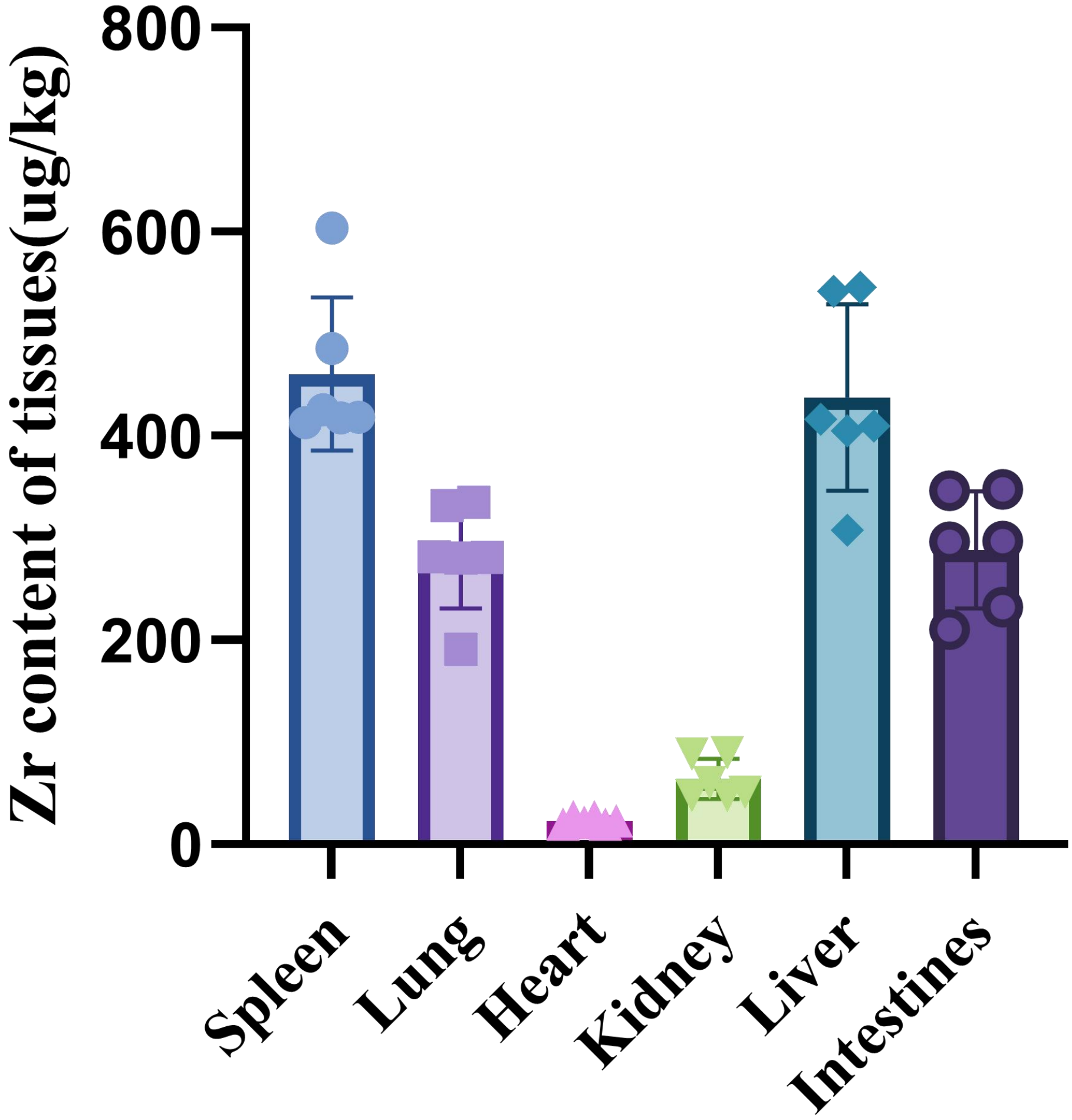

Figure S21 Biodistribution of MOF-G3-MNase in major organs after intraperitoneal administration. Zr contents in major organs at 24 h after intraperitoneal administration of MOF-G3-MNase, as determined by ICP-MS. MOF-G3-MNase was mainly distributed in the spleen, liver, and lung, with relatively lower levels detected in the heart and kidney. Data are presented as mean ± SD.
